# Unraveling Key Metabolomic Alterations in Wheat Embryos Derived from Freshly Harvested and Water-Imbibed Seeds of Two Wheat Cultivars with Contrasting Dormancy Status

**DOI:** 10.3389/fpls.2017.01203

**Published:** 2017-07-12

**Authors:** Aayudh Das, Dea-Wook Kim, Pramod Khadka, Randeep Rakwal, Jai S. Rohila

**Affiliations:** ^1^Department of Plant Biology, University of Vermont, Burlington VT, United States; ^2^Department of Biology and Microbiology, South Dakota State University, Brookings SD, United States; ^3^National Institute of Crop Science, Rural Development Administration Wanju-gun, South Korea; ^4^Faculty of Health and Sport Sciences, University of Tsukuba Tsukuba, Japan

**Keywords:** wheat, metabolomic profile, pre-harvest sprouting, PHS, raffinose, oligosaccharide, oxalate

## Abstract

Untimely rains in wheat fields during harvest season can cause pre-harvest sprouting (PHS), which deteriorates the yield and quality of wheat crop. Metabolic homeostasis of the embryo plays a role in seed dormancy, determining the status of the maturing grains either as dormant (PHS-tolerant) or non-dormant (PHS-susceptible). Very little is known for direct measurements of global metabolites in embryonic tissues of dormant and non-dormant wheat seeds. In this study, physiologically matured and freshly harvested wheat seeds of PHS-tolerant (cv. Sukang, dormant) and PHS-susceptible (cv. Baegjoong, non-dormant) cultivars were water-imbibed, and the isolated embryos were subjected to high-throughput, global non-targeted metabolomic profiling. A careful comparison of identified metabolites between Sukang and Baegjoong embryos at 0 and 48 h after imbibition revealed that several key metabolic pathways [such as: lipids, fatty acids, oxalate, hormones, the raffinose family of oligosaccharides (RFOs), and amino acids] and phytochemicals were differentially regulated between dormant and non-dormant varieties. Most of the membrane lipids were highly reduced in Baegjoong compared to Sukang, which indicates that the cell membrane instability in response to imbibition could also be a key factor in non-dormant wheat varieties for their untimely germination. This study revealed that several key marker metabolites (e.g., RFOs: glucose, fructose, maltose, and verbascose), were highly expressed in Baegjoong after imbibition. Furthermore, the data showed that the key secondary metabolites and phytochemicals (vitexin, chrysoeriol, ferulate, salidroside and gentisic acid), with known antioxidant properties, were comparatively low at basal levels in PHS-susceptible, non-dormant cultivar, Baegjoong. In conclusion, the results of this investigation revealed that after imbibition the metabolic homeostasis of dormant wheat is significantly less affected compared to non-dormant wheat. The inferences from this study combined with proteomic and transcriptomic studies will advance the molecular understanding of the pathways and enzyme regulations during PHS.

## Introduction

Pre-harvest sprouting (PHS) in wheat refers to the germination of physiologically mature seeds inside the spike, but before the crop is harvested (Sorrells and Sherman, 2007; Shu et al., 2015). It occurs due to periods of rainfall that cause excess humidity and warm temperatures in the field during seed maturation. Post-anthesis, wheat grains achieve maximum mass within a month, and then they undergo drying phase with rapid and gradual water loss and continue their ripening process. As a result, the endosperm cells succumb, and the embryo becomes dormant during this period (Thomason et al., 2009), which helps to prevent untimely germination of freshly matured seeds during short spells of conditions in fields that may even be very much favorable for seed germination (Née et al., 2017). During the post-green revolution period, for uniform and rapid seed germination at sowing wheat breeders have directly or indirectly applied genetic selections in the development of modern varieties that could have resulted in lowering the dormancy levels in wheat, and thus probably have decreased tolerance of wheat to PHS. Occurrence of PHS in wheat is determined by several factors, mainly: (i) inherent dormancy levels, (ii) duration and severity of humidity in the field, (iii) field temperature during high humidity period, (iv) growth phase of the maturing grain, and (v) spike morphology (Singh et al., 2014; Tuttle et al., 2015). The prevalence of PHS in the same cultivar could fluctuate from year-to-year depending on climatic conditions. Typical characteristics of PHS include swelling of the grain, splitting of the seed coat, discoloration of grain, and emergence of root and shoot (Thomason et al., 2009). PHS adversely affects milling properties, grain quality, seed viability and seedling vigor (Morgan, 2005). Pre-harvest germination of seeds causes degradation of protein and starch in endosperm and as a result the quality of wheat grains is reduced, which ultimately produces low-quality flour (Groos et al., 2002; Fakthongphan et al., 2016). PHS confines the end-use applications of wheat due to this downgraded grain and flour quality. A decreased test weight of the harvested crop is also a result of sprouting and is triggered by the conversion of starch to glucose by the α-amylase enzyme (Mares and Mrva, 2014; Kondhare et al., 2015). In China, the world’s largest wheat producer, almost 25 million hectares of wheat are affected by PHS (Liu et al., 2016); thus putting the PHS on a list that causes more than $1 billion loss per year to farmers on a global basis.

Seed dormancy level and seed germination rate are the two key processes involved in PHS, which are largely regulated by a series of complex biochemical processes in seed, and are controlled by genetic factors with substantial environmental influences (Koornneef et al., 2002; Nonogaki et al., 2010). Recent progress in functional genomics approaches such as transcriptomics, proteomics, and metabolomics have enhanced our limited knowledge of PHS (Gao and Ayele, 2014; Dong et al., 2015). Imbibition of dry seed reestablishes the molecular processes and eventually breaks the seed dormancy (Manz et al., 2005). Transcriptomics and proteomics studies in plants have also increased our current understanding on the dynamic relationship between transcripts and proteins during seed maturation and their amendments after imbibition (Nakabayashi et al., 2005; Chibani et al., 2006; Holdsworth et al., 2008). A comparative proteomic analysis was carried out by Kamal et al. (2009) in seeds of PHS-resistant (cv. Keumgang) and PHS-susceptible (cv. Jinpum) wheat. The researchers identified 73 differentially expressed proteins between the resistant and susceptible cultivar, which fall under nine broad functional categories: metabolism, storage, photosynthesis, amino acid, allergy, stress, protein synthesis, enzyme, and hypothetical protein. Another proteomic study consisting of a transgenic PHS-resistant wheat [reduced Thioredoxin h (trx h) expression] and wild-type PHS-susceptible wheat (cv. Yumai 18), after 48 h of imbibition, identified 16 differential abundant proteins (Guo et al., 2011). They suggest that trx h gene, along with other proteins such as Serpin, 14-3-3, and WRKY6 transcription factor might be a key determinant for seed germination. It possibly will increase the activity of thiocalsin by reducing disulfide groups and inhibiting Serpin, and might be playing a role during PHS in wheat especially by promoting proteins degradation. Recently, transcriptomic investigations of dormant and non-dormant pea seeds has identified 148 differentially expressed genes (Hradilová et al., 2017). Metabolomic analysis of separated seed coats from pea genotypes revealed significantly higher contents of proanthocyanidins (dimer and trimer of gallocatechin), quercetin, and myricetin rhamnosides and hydroxylated fatty acids in dormant compared to non-dormant seeds. The transcriptomic studies have also demonstrated that various transcription factors are differentially expressed in wheat during seed development and first 48 h of post-imbibition germination (Wilson et al., 2005; Wan et al., 2008). The authors suggest that the majority of the transcripts required for germination after imbibition may accumulate in the embryo during seed maturation or at least prior to germination. Abscisic acid (ABA) has also been reported to be associated with seed dormancy. Gao et al. (2012) meticulously performed comparative transcriptomic analysis between dormant and after-ripened seeds in both dry and water-imbibed states. The study found a total of 3067 probesets, grouped into 16 clusters, which show differential expression of fivefolds or higher. Of these, only 58 probesets showed differential expression between dry after-ripened and dormant seeds, and 36 probesets were found to be regulated by dormancy only. Overall, this microarray study identified several biological processes such as: jasmonate biosynthesis, cell wall modifications, epigenetic mechanisms that could be critical in germination of water-imbibed, after-ripened wheat seeds when dormancy status is at low level in wheat seeds. An excellent review by Nonogaki (2014) on seed dormancy and seed germination research has highlighted a complex biological fact that there is a risk of over-simplifying molecular functions as positive and negative regulators because a single gene product could employ a negative feedback while serving as a positive regulator. Quantitative trait loci (QTL) analyses in wheat have shown that seed dormancy is regulated by major loci on chromosome 4A (Shorinola et al., 2016; Torada et al., 2016), chromosomes 5D, 3A, and 3D (Zhou et al., 2017). Approaches using map-based cloning have revealed that the TaMFT gene in wheat directly affects PHS through seed dormancy (Liu S. et al., 2013). Moreover, TaSdr genes were established to be key regulators of seed dormancy and PHS (Zhang et al., 2014). These evidences and more detailed discussions elsewhere such as by Zhou et al. (2017) suggest that either loss or reduced seed dormancy are the two key regulatory factors to determine the PHS phenotype in wheat. It has also been suggested that the occurrence of pre-harvest α-amylase in wheat seeds points toward the fluctuation of major plant hormones, including abscisic acid (ABA) and gibberellins (GAs), which play key roles in grain development. In the past two decades, research has been focused on breeding PHS-resistant varieties through identification of QTLs that confer PHS tolerance to wheat (Kulwal et al., 2004, 2005; Lohwasser et al., 2005; Torada et al., 2005; Chen et al., 2008; Gao et al., 2013; Cao et al., 2016; Shorinola et al., 2016), but very little is known at the level of metabolome. The metabolome is downstream of transcriptome and proteome, and cannot be predicted directly by the genomic information (Hong et al., 2016); and one of its attraction is in providing reliable molecular markers because metabolites are dynamically and comparatively closest to the observed phenotypes compared to the transcripts and proteins. Further, metabolomics studies can complement the insights drawn from transcriptomics and proteomics studies and may suggest a direct link between a gene and the function of the metabolic network (Fiehn et al., 2000). On a molecular breeding assignment the QTL mapping relies heavily on precise phenotyping of the trait, and metabolite profiling is proficient to provide a novel and precise phenotyping tool to plant breeders in the form of metabolite variants. Thus the developments in metabolomics offer great promise to plant breeding programs by developing metabolic markers, metabolic quantitative trait loci (mQTL), as well as to basic biology programs by contributing substantial knowledge toward the understanding of various cellular mechanisms (Nambara and Nonogaki, 2012; Herrmann and Schauer, 2013).

Metabolomics is comparatively a new branch of high-throughput functional genomics that has the potential to uncover the differential accumulation of metabolites or small molecules at global level within a cell at a given time (Weckwerth, 2010). Global metabolomics via the use of mass spectrometry (MS) permits the identification of 1000s of metabolites associated with a specific treatment (Patti et al., 2012). Metabolomics delivers an improved gateway of measuring biochemical activity directly by monitoring the substrates and products that are converted throughout the cellular metabolism of the plant (Saito and Matsuda, 2010). Quantitative measurements of the metabolites in the cell convey an extensive outlook of the functional status of the plant tissue, which can be useful for the assessment of gene functions (Ramalingam et al., 2015). A global metabolomics approach also provides a powerful tool to study the temporal regulation of various metabolic pathways. Although the effects of PHS conditions on wheat have been the subject of intense research at the levels of quantitative genetics, physiology, molecular biology, and genomics, no detailed metabolomics-based study has been performed yet on wheat embryos related to PHS and/or seed dormancy (Zanetti et al., 2000; Kulwal et al., 2004; Carvalho and Beleia, 2015; Shu et al., 2015). Recent studies have started to emerge on infrared spectroscopy-based metabolomic profiling, which assesses the length of the germination process in barley and wheat (Burke et al., 2016). A recent excellent report recommended using the near-infrared spectroscopy to the grain industry to detect instances of germination in cereal seeds at a very early stage where symptoms of germination are not visible to human eyes, and thus barely sprouted grains can be noticed (Jones, 2017). Moreover, another study by Liu et al. (2016) used the metabolomic approach to define various metabolites from 20-days-post-anthesis to after 30-days-post-harvest ripening which were induced by introduction of *anti-trx-s* gene in wheat that facilitate PHS-resistance. They noticed that in freshly matured seeds, most metabolites of glycolysis, TCA cycle, choline metabolism, biosynthesis of proteins, nucleotides and fatty acids were at significantly lower levels in transgenic than in wild-type wheat.

We hypothesized that if the PHS-tolerant and PHS-susceptible wheat genotypes are water-imbibed, they may show differential accumulation of metabolites in the excised embryos, and this knowledge may shed light on germination and PHS mechanisms. To identify dormancy-associated metabolomic alterations in wheat embryos that are also associated with PHS, we carried out metabolomic profiling of two Korean wheat cultivars (*Triticum aestivum* L.) that possess contrasting phenotypes for PHS in the field; Sukang is a PHS-tolerant cultivar (PHS rate of 0.2% compared to a check variety ‘Keumkang,’ which has a PHS rate of 30.4%) and Baegjoong is PHS-susceptible cultivar (PHS rate of 23.9% compared to ‘Keumkang’) (Park et al., 2008, 2009). Parents of the two cultivars are genetically distant, but they show similar agronomical characteristics for growth duration, date of heading and maturity dates (Park et al., 2008). In terms of flour characteristics, the two varieties have different levels of protein content but similar characteristics in the high molecular weight glutenin subunit (HMW-GS) composition (Park et al., 2009). The objective of this study was to compare metabolomic profiles of embryos from the water-imbibed wheat seeds, and find key processes where the two wheat cultivars, which contrast in PHS tolerance after imbibition, differ and by data analyses and previous reports to get insights into the metabolomic activities of PHS-tolerant cv. Sukang over PHS-susceptible cv. Baegjoong. An in-depth metabolomic insight can complement the knowledge gained from other fields such as plant physiology, genetics, transcriptomics and proteomics to elucidate the physiological and biochemical mechanisms involved in PHS tolerance in wheat as well as in other cereals (Das et al., 2015, 2016).

A metabolomic analysis of primary metabolites using liquid chromatography (LC) and gas chromatography (GC) coupled with MS allowed us to identify differentially expressed metabolites (such as carbohydrates, amino acids, lipids, oxalate, different plant hormones and their precursors) in response to water-imbibition of dormant and non-dormant wheat seeds. This study offers new knowledge for facilitating the development metabolite markers that can be utilized along with DNA markers to design PHS-resistant wheat varieties in future.

## Materials and Methods

### Plant Materials

Two wheat cultivars Sukang (PHS-tolerant) and Baegjoong (PHS-susceptible) were grown in a field experiment with three replicated plots in a complete randomized design. Spikes of each wheat cultivar were harvested at physiological maturity (Calderini et al., 2000), and air-dried at room temperature until seed moisture content reached approximately 12%. After hand-threshing, morphologically sound seeds that show similar size, color, health, and maturity levels were selected and surface sterilized with 50% bleach solution. Fifty seeds from each replication were placed crease down in Petri dishes (90 mm diameter) with two layers of Whatman No. 3 filter paper with 6 ml of sterile distilled water and incubated at 20°C for 48 h in dark (Nyachiro et al., 2002). For this investigation, we selected 48 h time point for imbibition based on Yu et al. (2014) where they have described three distinct phases during seed germination process that commences with imbibition: a rapid initial uptake phase (0–12 hours after imbibition [HAI]), a plateau phase (12–24 HAI), and a further water uptake phase (24–48 HAI). After imbibition treatment, all the seeds were lyophilized. Embryos from more than 120 seeds were isolated manually with the help of a scalpel and ground in liquid nitrogen using a mortar and pestle. The pulverized material was stored at -80°C until metabolomic profiling.

### Metabolomic Profiling

The metabolomic profiling in this study was focused on wheat embryos harvested at 0 and 48 h time points. The embryonic tissue samples were submitted to the Metabolon, Inc. (Durham, NC, United States). The sample preparation and analysis process was carried out essentially as described previously (Evans et al., 2009; Oliver et al., 2011; Tripathi et al., 2016; Das et al., 2017). Briefly, the samples were extracted in 400 μl of methanol using an automated liquid handling system, and the resulting samples were split into independent aliquots for analysis on three MS instrument platforms: GC/MS and two UPLC/MS platforms- where one was optimized for positive ionizations, and another platform was optimized for negative ionizations. Three controls were also prepared with the experimental samples: (i) pooled samples from three biological replications served as one technical replicate; (ii) ultra-pure water was used as a blank; (iii) a cocktail of quality control (QC) standards that included pooled samples, ultra-pure water and an aliquot of solvents used for extraction. Instrumentation derived variability was determined by calculating the median relative standard deviation (RSD) for the standards and overall process variability was calculated by measuring the median RSD for all metabolites present in the pooled matrix.

As described by Evans et al. (2009) in details, the Metabolon’s LC/MS portion of the platform was based on a Waters ACQUITY ultra-performance liquid chromatography (UPLC) and a Thermo Scientific Q-Exactive high resolution/accurate mass spectrometer interfaced with a heated electrospray ionization (HESI-II) source and the Orbitrap mass analyzer was operated at 35,000 mass resolution. The dried sample extract were reconstituted in acidic or basic LC-compatible solvents and were divided into three aliquots. First aliquot was analyzed using acidic positive ion optimized conditions, and were eluted from C18 column (Waters UPLC BEH C18-2.1 mm × 100 mm, 1.7 μm) using water and methanol containing 0.1% formic acid. Second aliquot was analyzed using basic negative ion optimized conditions, were eluted from separate but similar C18 column using methanol and water with 6.5 mM ammonium bicarbonate. The third aliquot was analyzed via negative ionization following elution from a HILIC column (Waters UPLC BEH Amide 2.1 mm × 150 mm, 1.7 μm) using a gradient consisting of water and acetonitrile with 10 mM ammonium formate. The MS analysis alternated between MS and data-dependent MS^2^ scans using dynamic exclusion, and the scan range was from 80 to 1000 mass to charge ratio (*m/z*).

The same embryonic samples were also derivatized under dried nitrogen using bistrimethyl-silyltrifluoroacetamide and analyzed by GC-MS. Derivatized samples were separated on a 5% diphenyl/95% dimethyl polysiloxane fused silica column (20 m × 0.18 mm ID; 0.18 μm film thickness) with helium as carrier gas and a temperature ramp from 60° to 340°C in a 17.5 min period. The samples were analyzed on a Thermo-Finnigan Trace DSQ fast-scanning single-quadrupole mass spectrometer using electron impact ionization (EI) and operated at unit mass resolving power. The scan range was from 50 to 750 m/z. The MS data was normalized and the metabolite quantification was performed by calculating the area-under-the-curve. Further, the MS peaks were also processed for QC using Metabolon’s hardware and software. For peak identification the Metabolon maintains a library based on authenticated standards that contains the retention time/index (RI), mass to charge ratio (*m/z)*, and chromatographic data including MS/MS spectral data. Compounds were identified by comparisons to Metabolon’s library entries of purified standards (Dehaven et al., 2010) and their recurrent unknown entities. The biochemical identifications in this study were based on three criterion: retention index within a narrow RI window of the proposed identification, accurate mass match to the library +/- 0.005 amu, and the MS/MS forward and reverse scores between the experimental data and authentic standards. The MS/MS scores are based on a comparison of the ions present in the experimental spectrum to the ions present in the library spectrum.

### Statistical and Computational Analysis

Since all the treatments were comprised of three replicates (*n* = 3 for all groups), the statistical significance of the results was evaluated using Welch’s two sample *t*-test and a level of significance of *p* ≤ 0.05 for two group comparisons. The matched pair *t*-test was used to test whether two unknown means are different from paired observations from the same subjects. Principal component analysis (PCA), which is an unsupervised analysis and reduces the dimension of the data, was performed using R 3.3.1 software^1^ to reveal the variability and significance of the dataset that can show- how the metabolites are differentially expressed in Sukang and Baegjoong seeds when imbibed in water.

For the clustering of the data, the log2-transformed accumulation values of the metabolites were used, and the heat map included the hierarchical clustering of the metabolites using Gene Cluster 3.0 software (de Hoon et al., 2004). The clusters were visualized using JAVA TREEVIEW software (Saldanha, 2004). MapMan application software (MapMan Version 3.5.1R2) was used to understand the metabolic distribution and metabolic regulation in response to PHS. Different metabolic networks were portrayed using the MetScape 3.1 plugin for Cytoscape software, version 3.0.1, and for ID referencing, we used the KEGG database (Kanehisa and Goto, 2000; Shannon et al., 2003; Karnovsky et al., 2012). The data analysis was done using R package ggplot2 (Wickham, 2016).

## Results and Discussion

### PHS Phenotype and Differential Accumulation of Metabolites in Sukang and Baegjoong in Response to Water-Imbibition

**FIGURE 1:**
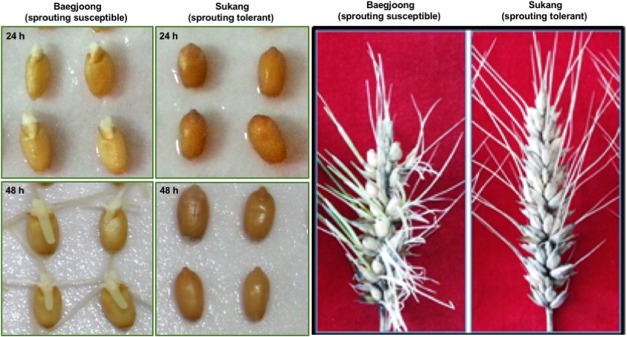
The phenotypic differences of Baegjoong and Sukang after imbibition in water. The seeds of both cultivars used in this experiment were freshly harvested. **Left panel** shows Baegjoong and Sukang seeds after 24 and 48 h of imbibition. The **Right panel** shows the phenotype of the whole spike.

Before examining the differential accumulation of metabolites in the embryos of Sukang and Baegjoong, we checked the sprouting behavior of the two cultivars. **Figure 1** shows the phenotypic differences between Sukang and Baegjoong after 24 and 48 h of water-imbibition. Early seed germination was observed in Baegjoong at 24 h of imbibition, whereas Sukang remained dormant even after 48 h of imbibition. Earlier physiological studies had also shown the pre-mature germination in Baegjoong compared to Sukang (Park et al., 2008, 2009).

**FIGURE 2:**
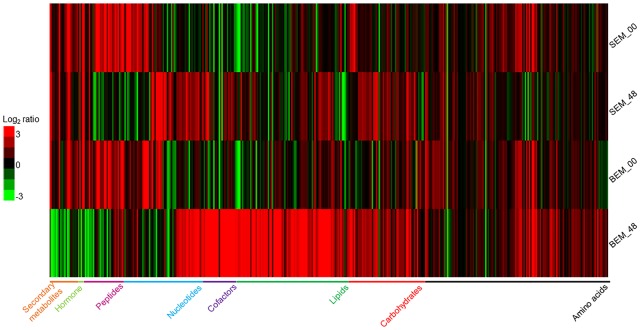
The heat map shows the differential expression of metabolites in wheat embryos at two different time points (0 and 48 h) of imbibition as detected by LC/GC–MS. [S-Sukang; B-Baegjoong; and EM-embryo]. Heat map was developed using Gene Cluster 3.0 and visualized by using JAVA TREEVIEW.

The analyses of the metabolomic profiling data of isolated embryos from these imbibed seeds showed that in response to water-imbibition, the metabolites were differentially accumulated between the two varieties at both time points (**Figure 2** and Supplementary Table S1). The metabolomic profiling revealed a concurrent detection of 409 metabolites that fall under several broad functional categories, such as carbohydrates, amino acids, lipids, cofactors, nucleotides, peptides, and secondary metabolites. Supplementary Figure S1 shows a PCA plot of all of the sample’s metabolites. The two cultivars were widely separated at the analyzed time points due to the general differences in the abundance of metabolite levels. However, it is important to note that for overall metabolite profiling, the Baegjoong embryo at 0 h (BEM-00) is similar to the Sukang embryo at 0 h (SEM-00), but the SEM-00 and BEM-00 were clearly differentiated from the Sukang embryo at 48 h (SEM-48) and the Baegjoong embryo at 48 h (BEM-48), respectively.

The disparity in the general number of metabolites in Baegjoong was apparent in the strong skewing of the statistical results when the cultivars were compared to each other (**Figure 2**). In this study, 409 metabolites were found to be significantly accumulated between the two cultivars, and the majority of metabolites were significantly higher in Baegjoong. On the other hand, it seems very likely that the metabolites, whose abundance was relatively higher in Sukang (such as lipids: glycerophosphorylcholine, 1-linoleoyl-GPI, 1-palmitoyl-GPA, glycerol 3-phosphate, 1-oleoyl-GPA, and 1-linoleoyl-GPS; raffinose family of oligosaccharides (RFO’s): fructose, galactitol, mannitol, mannose, and myo-inositol; and secondary metabolites like ferulate) might be notable for PHS-resistance. It is also to be noted that overall mean value for the accumulated metabolites did not change in Sukang in response to 48 h of imbibition, whereas Baegjoong showed a significantly higher mean value for the accumulated metabolites (Supplementary Figure S2), probably due to significant changes in the metabolic homeostasis of Baegjoong. A MapMan (Thimm et al., 2004) based analysis (Supplementary Figures S3A,B) mapped 101 metabolites out of 409, and it showed a clear difference between the two varieties in various metabolic processes that were affected by the water-imbibition of wheat seeds.

### Lipid and Oxalate Metabolism during PHS

**FIGURE 3:**
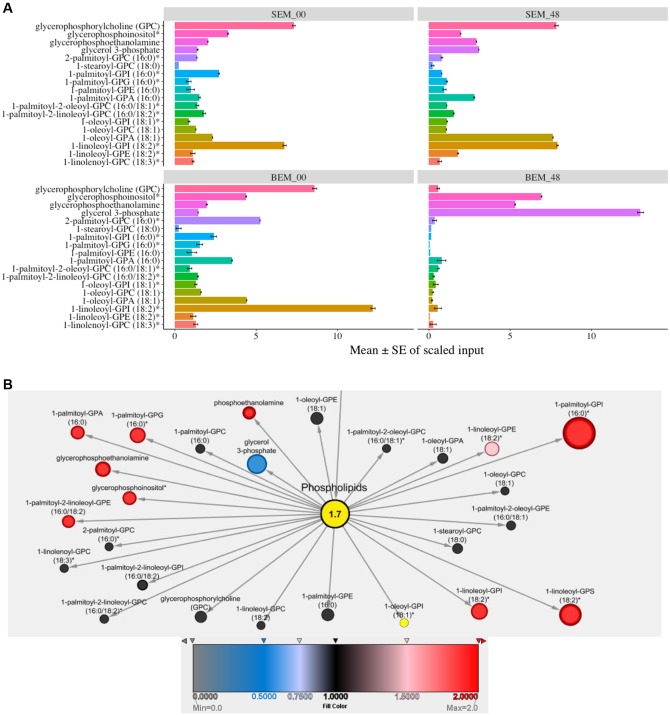
Differential expression of lipids. **(A)** Mean values ± SE of scaled input of membrane lipids in SEM compared to BEM at 48 and 0 h. Error bars represent standard error (SE), data was accumulated from three replicates. For *p*-values see Supplementary Table S2 [S-Sukang; B-Baegjoong; and EM-embryo]. **(B)** Lipidomic interactions of all the input candidates in the study are shown using MetScape 3 software, along with their predicted interaction partners where the phospholipid has a centralized place.

Cellular membranes are an integral part of cell organelles. Phospholipids are the backbone of the cellular membranes and can serve as a precursor for the generation of secondary lipids during many developmental processes (Fuller, 2006). Interestingly, at 48 h, most of the phospholipids were highly expressed in Sukang, and comparatively very few in Baegjoong. Baegjoong showed decreasing levels of membrane lipids and lysolipids between the 0 and 48 h time window (**Figure 3A** and Supplementary Table S2). At the same time, Sukang showed increasing levels of many lysolipids. The pattern suggests membrane instability and/or improperly timed membrane turnover/apoptosis during seed maturation of Baegjoong, a PHS-susceptible cultivar. A structurally diverse range of plant lipids, including fatty acids, contributes to a range of biological processes, such as membrane structure, primary and secondary metabolism, and extracellular and intracellular signaling in plants (Horn and Chapman, 2014). Lipid metabolism greatly affects seed development, dormancy, and germination (Penfield et al., 2007). Defects in β-oxidation can affect the seed dormancy levels of seeds, and, as determined from the metabolite levels in this study, the β-oxidation process in Baegjoong is significantly enhanced, which could have resulted in oxidation of many fatty acids. Moreover, higher levels of lipid peroxidation cause membrane damage (Wang et al., 2012), and this proposition is also supported in our analysis by compromised levels of most of the phospholipids, e.g., oxylipins and glycerolipids. Similar to other abiotic stressors, pre-mature seed germination may also induce the production of reactive oxygen species (ROS) and other cytotoxic compounds (Bailly et al., 2008). These stress-induced compounds could further lead to the breakdown of lipids.

We also found that in response to water-imbibition, there is an elevation of 18:1 (cis-vaccenate, oleate, linoleate, and linolenate) polyunsaturated fatty acids in Sukang. It is known that phospholipase A2 (PLA_2_) mediates signaling cascades during developmental processes (Verlotta et al., 2013) and increased levels of free fatty acids, such as linoleate, linolenate, and palmitate, suggests that this reversal effect for pre-mature germination is mediated by increased PLA_2_ activity, which is steadily detected in Baegjoong (Supplementary Figure S4 and Table S1).

From a systems biology perspective, and for a better understanding of functional lipidomic interactions occurring in cell during PHS, we used Metscape to look through an overall scenario of all of the phospholipids and fatty acids during PHS. **Figure 3B** shows the putative lipidomic interaction of all of the input candidates in this study as well as their predicted partners. This study revealed various carbohydrates participating in glycolysis, TCA cycle, pentose phosphate pathway and other significant biological processes as well as functionally interacting with related compounds, which would eventually exert an effect on the biological and cellular processes when the wheat seeds are exposed to high humidity through rains in field or water-imbibition in lab experiments.

**FIGURE 4:**
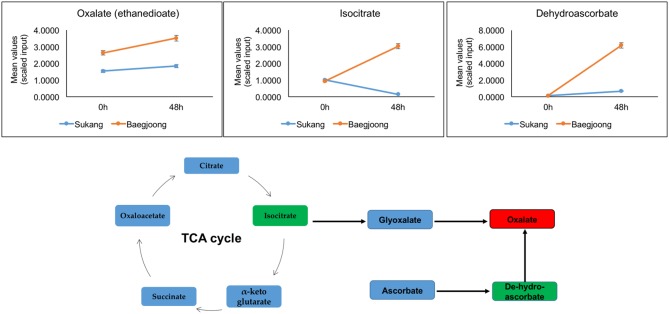
Differential expression of oxalate (red box) and its precursors (green box) in Sukang and Baegjoong. The figure shows that at 0 and 48 h the oxalate and its precursors are elevated in Baegjoong, but not in Sukang. Error bars represent ± SE, data was accumulated from three replicates.

In Baegjoong, one compound, oxalate, stands out as showing the largest cultivar-related difference. Oxalate is a dianion that is synthesized by partial oxidation of the carbohydrates in plants (Osmond, 1967), and could be one of the key metabolites playing a crucial role during seed germination. In this metabolomic profiling, at 0 and 48 h, oxalate shows a major alteration between the two cultivars (**Figure 4**). Oxalate formation can be induced by metabolic precursors, such as glyoxylate, isocitrate, oxaloacetate, and ascorbate (Nakata, 2003; Xu et al., 2006). During developmental processes, oxalate oxidase can be involved in the production of ROS in plant tissues (Voothuluru and Sharp, 2013; Baxter et al., 2014). Germin (a type of oxalate oxidase), which is a major protein in wheat embryos and a marker for seed germination, seems to be involved in regulating seed dormancy status of the seed (Caliskan and Cuming, 1998). Berna and Bernier (1999) showed that the expression of wheat germin gene is stimulated during seed germination. In wheat root tips, a high level of oxalate oxidase expression generates excess amount of H_2_O_2_, which induces cell death and is required for cell wall crosslinking (Caliskan and Cuming, 1998; Berna and Bernier, 1999; Delisle et al., 2001; Baxter et al., 2014). Thus, comparatively higher abundance of oxalate in Baegjoong may suggest a lack of oxalate oxidase activity or an increased level of lipid degradation in this cultivar. It has been suggested that oxalate catabolism in *Arabidopsis* may determine the fate of seed germination (Foster et al., 2012). Based on the previous findings and by correlating our metabolic profiling derived results, we expect that a higher level of oxalate in Baegjoong may have induced seed germination after imbibition; therefore, sprouting occurs rapidly when seed encounters high humidity in field. In contrast, a comparatively lower level of oxalate in Sukang, may have delayed the seed germination and can prevent PHS.

### Differential Accumulation of Hormone and Hormone-Related Compounds

**FIGURE 5:**
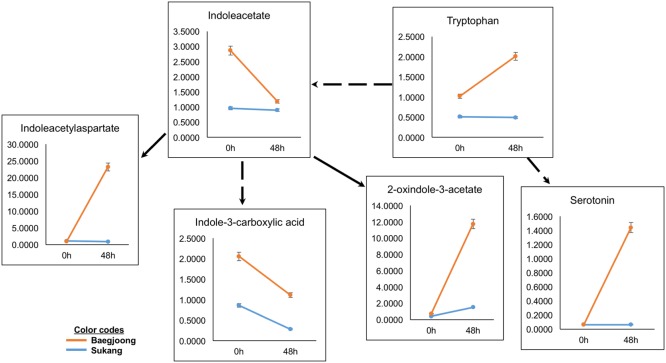
Differential expression of auxin-related compounds in Sukang and Baegjoong after 48 h of imbibition. Broken arrow emphasizes that more than one step is involved in the process. Error bars represent ± SE, data was accumulated from three replicates.

Auxins (e.g., indole-3-acetic acid, IAA) and amino acids (e.g., tryptophan), play key roles in balancing seed dormancy and seed germination rates (Ramaih et al., 2003). Metabolomic profiling of the two cultivars detected variable abundance of many auxin-related compounds in Baegjoong in the 48 h samples (**Figure 5**). Interestingly, the precursor amino acid tryptophan was induced showing higher accumulation at 48 h after water-imbibition, whereas Sukang did not show any significant changes. Indoleacetate abundance did not change much in the Sukang samples but showed a steep reduction in Baegjoong at 48 h after water-imbibition. Three IAA catabolites were also detected, including indole-3-carboxylate, which showed a similar pattern at 48 h to IAA but with two other metabolites (indoleacetyl-aspartate and 2-oxindole-3-acetate) showing much higher levels in Baegjoong at 48 h time point. Serotonin, another potential regulatory molecule, which acts as a natural auxin, exhibited a similar pattern (**Figure 5** and Supplementary Table S3) (Pelagio-Flores et al., 2011).

**FIGURE 6:**
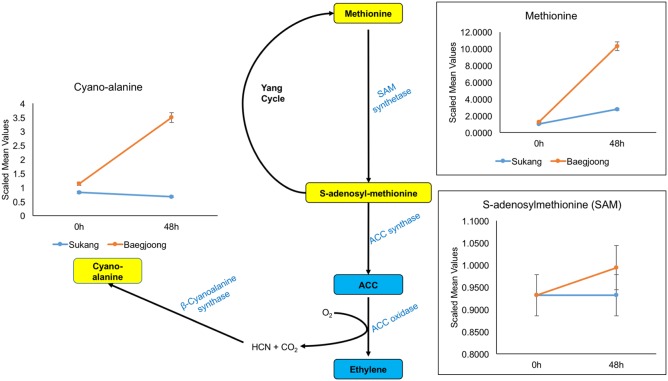
Differential expression of cyano-alanine and ethylene precursors in Sukang and Baegjoong after imbibition in water. The figure demonstrates that methionine and *S*-adenosyl-methionine, which are involved in ethylene biosynthesis and the Yang cycle, are differentially expressed after imbibition (48 h) in both cultivars [ACC, 1-aminocyclopropane-carboxylate]. Error bars represent ± SE, data was accumulated from three replicates.

Ethylene is one of the key plant hormones that controls dormancy in various plants (Corbineau et al., 2014). Cyano-alanine is a co-product of ethylene biosynthesis (Yip and Yang, 1988, 1998). Consistent with previous reports, our metabolomic profiling shows that after water-imbibition of seeds, there is no change in abundance for cyano-alanine in Sukang embryos, but over time, it increased in Baegjoong embryos (**Figure 6** and Supplementary Table S3). Additionally, methionine and *S*-adenosyl-methionine, which are involved in ethylene biosynthesis and the Yang cycle, are also abundant in the Baegjoong embryos but accumulated at reduced levels in Sukang. Several reports indicate that ABA maintains seed dormancy in accordance with other hormones, genes and metabolites in plants, such as wheat and *Arabidopsis* (Liu X. et al., 2013; Martinez et al., 2016). Walker-Simmons (1987) compared the sprouting-susceptible and sprouting-resistant wheat cultivars and found a 25% lower ABA level in sprouting-susceptible cultivars compared to sprouting-resistant cultivars, which indicated the potential of ABA in inhibiting embryonic germination effectively in the sprouting-resistant cultivar. In our metabolic profiling, putatively detected ABA had a very small peak and thus could not be confirmed with statistical significance. However, ions believed to come from ABA were found only in the first time point of the Sukang samples (none at 48 h), and were not detected at all in Baegjoong samples at any time points (Supplementary Figure S5). Additionally, two major ABA precursors, i.e., mevalonate and mevalonolactone, are higher at 0 h in Sukang and were gradually reduced following the water imbibition treatment. In contrast, for Baegjoong, both of these ABA precursors are accumulated at much lower levels (Supplementary Figure S5).

It has been reported that in potato, ethylene treatment rapidly inhibits sprout growth in a completely reversible manner (Rylski et al., 1974). Specifically, auxin-treated tubers show dose-dependent enhancement of ethylene production and an inhibition of sprout growth (Suttle, 2003). Although, ethylene promotes germination in dormant seeds, it does so in cooperation with ABA, which is the primary facilitator of seed dormancy (Koornneef et al., 2002; Gubler et al., 2005). Our metabolic profiling also elucidates that how ethylene precursors and ethylene-related metabolites are regulated in response to imbibition in these two wheat cultivars. Because ethylene is considered to be an aging hormone, a higher level of ethylene expression might induce senescence (Schaller, 2012), and in Baegjoong, it might be signaling an after-ripening effect to trigger the PHS (Carrera et al., 2008). In this study, ABA-mediated responses during PHS show a similar result to previous reports (Kashiwakura et al., 2016). It is evident that ABA can be directly synthesized from trans-farnesyl pyrophosphate or isopentenyl diphosphate (IPP) from mevalonic acid (Rohmer et al., 1993; Hirai et al., 2000; Milborrow, 2001). Thus, the observed enhancement of mevalonate and mevalonolactone levels at 48 h in Baegjoong (Supplementary Figure S5) suggests that water-imbibition might be inducing seed sprouting subsequently, which may lead to PHS in field. Although, it is also true that ABA synthesis is similarly regulated downstream of the mevalonate pathway mainly at steps catalyzed by NCED (9-cis-epoxycarotenoid dioxygenase) and ABA8′OH (ABA8′ hydroxylase), but it does not reflect in our findings (Seiler et al., 2011; Finkelstein, 2013).

### Alterations in the Carbohydrate and Amino Acid Metabolism

**FIGURE 7:**
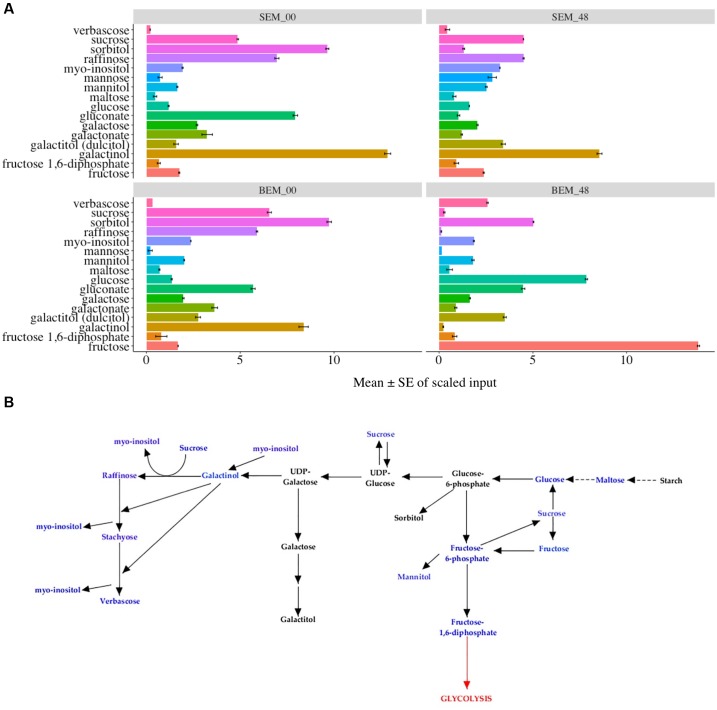
Differential expression of carbohydrate pathways. **(A)** Mean value ± SE in the raffinose family oligosaccharides (RFOs), which are involved in carbohydrate metabolism in SEM compared to BEM (0 and 48 h time points are shown) [S-Sukang; B-Baegjoong; and EM-embryo]. Error bars represent ± SE, data was accumulated from three replicates. See Supplementary Table S5 for *p*-values. **(B)** Differential expression of RFOs. Blue font indicates carbohydrates that were detected in the study, and black font indicates adjoining carbohydrates, based on KEGG database, in the same pathway. Error bars represent ± SE, data was accumulated from three replicates.

**Figures 7A,B** show the data and pathways associated with carbohydrate metabolism, including the RFO pathway (also see Supplementary Table S4). RFOs are abundant in plant seeds and are synthesized through the involvement of a set of galactosyltransferases that successively transfer galactose units from galactinol to sucrose. RFOs accumulate during seed development and promptly decrease during seed germination (Peterbauer and Richter, 2001). While most of these metabolites are higher in Baegjoong samples at 48 h, there are several exceptions that may be informative for future PHS experiments. First, sucrose and other metabolites of the RFO pathway (such as galactinol, raffinose, gluconate, and galactose) all start at much higher levels in Sukang samples but then decrease sharply at the 48 h time point. In contrast, glucose, fructose, maltose (breakdown product of starch), and verbascose levels increase at 48 h in Baegjoong samples. In other words, most of the major carbohydrates that are involved in RFO are greatly affected in the PHS-susceptible cultivar Baegjoong, but Sukang shows a constant level of carbohydrate expression in terms of hexose sugar. Simsek et al. (2014) used a scanning electron microscope, and observed intact starch granules in non-sprouted wheat varieties; but found that the starch granules are degraded in sprouted varieties due to the high α-amylase activity. Consistent with this finding, high level of breakdown products of starch, such as glucose, fructose, maltose, and verbascose were observed in Baegjoong embryos after 48 h of water-imbibition. In this study, we showed that individual hexose sugars, starches and their precursors in the RFO family are affected by 48 h of water-imbibition. The TCA cycle is a crucial respiratory pathway that is essential for energy delivery to different organelles and for the maintenance of various physiological functions (Fernie et al., 2004). Our study indicates that after imbibition, the abundance level of several metabolites of TCA cycle such as citrate, fumarate, isocitrate, malate, succinate, and cis-aconitate are significantly increased in the PHS-susceptible cultivar Baegjoong at 48 h compared to those in Sukang (Supplementary Figure S6). We believe that the increased level of metabolites of the TCA cycle in the Baegjoong could have a major impact on the energy synthesis with rapid sugar utilization, and thus it may have a correlating effect on the poor quality of wheat grains after imbibition. The exceptions to this include sucrose, hexose diphosphate isobar (fructose-1,6-diphosphate, F-1,6-DP), and three higher order oligosaccharides: the RFO verbascose, stachyose (tetrasaccharide of galactose units), and raffinose (tetrasaccharide of galactose, glucose, and fructose units). While a higher F-1,6-DP may indicate higher activity in the glycolytic pathway, and although other compounds in the pathway were not increased, the other metabolites are consistent with the observation that Baegjoong samples contains higher levels of polysaccharides.

**FIGURE 8:**
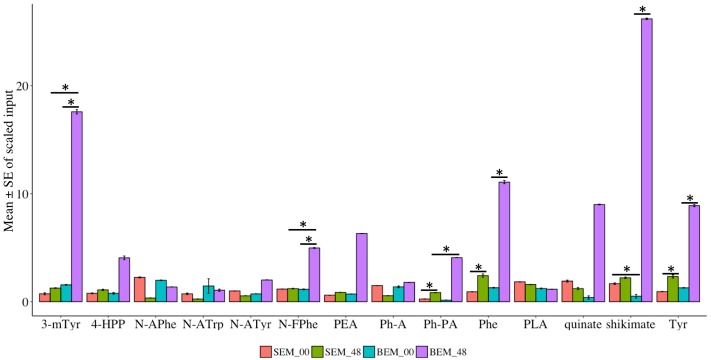
Metabolomic alterations in the precursor compounds of the aromatic amino acid biosynthetic pathway in Sukang and Baegjoong after 48 h of imbibition. Error bars represent ± SE, data was accumulated from three replicates. ^∗^Indicates *p* < 0.05, for more information see Supplementary Table S6. 4-HPP, 4-hydroxyphenylpyruvate; PLA, phenyllactate; *N*-FPhe, *N*-formylphenylalanine; *N*-APhe, *N*-acetylphenylalanine; *N*-ATrp, *N*-acetyltryptophan; *N*-ATyr, *N*-acetyltyrosine; 3-mTry, 3-methoxytyrosine; Ph-A, phenylacetate; Ph-PA, phenylpyruvate; PEA, phenethylamine; Phe, phenylalanine; Tyr, tyrosine; Trp, tryptophan.

Amino acids are fundamental compounds that independently or synergistically influence the physiological activities of plants (Causin, 1996). Most importantly, amino acids are building blocks for active plant proteins, which are involved in major cellular and molecular functions (Rai, 2002). Specifically, plant nitrogen metabolism is highly regulated by the major amino acids, which have a potential role in the assimilation of nutrients (Buchanan et al., 2015). Our metabolic profiling indicated that 137 metabolites related to either amino acids or their conjugates and metabolism are differentially expressed during imbibition in Baegjoong and Sukang (Supplementary Figure S7 and Table S5). These metabolites fall into one of the following categories: serine family phosphoglycerate-derived amino acids, aromatic amino acid (PEP-derived), aspartate family of amino acids (OAA derived), glutamate family of amino acids (alpha-keto-glutarate-derived), branched chain amino acids (both OAA-derived and pyruvate-derived), amines-polyamines and amino acids that are involved in glutathione metabolism. Globally, amino acid metabolism was highly altered in the PHS-susceptible cultivar Baegjoong compared to PHS-tolerant Sukang from the 0 to 48 h time points (Supplementary Figure S7). The present investigation showed that aromatic amino acid metabolism was induced in the PHS-susceptible Baegjoong at 48 h, whereas the changes were very little in the PHS-tolerant Sukang (**Figure 8** and Supplementary Table S6). Interestingly, shikimate, one of the precursor compounds of the aromatic amino acid biosynthetic pathway, was greatly induced in Baegjoong after 48 h of imbibition. The shikimate pathway has the potential to affect other biosynthetic pathways, such as tryptophan, tyrosine, and phenylalanine (Maeda and Dudareva, 2012). Affected aromatic amino acid metabolism may compromise essential precursors which are required for the synthesis of a wide range of secondary metabolites essential for various biological processes (Tzin and Galili, 2010). The oxidized form of cysteine and several other catabolites of amino acids fit this criterion. Phosphoenolpyruvate (PEP) is the main precursor of the aromatic amino acids, and chorismate is the branch point for the synthesis of major aromatic amino acids such as: tryptophan, tyrosine, and phenylalanine (Salter et al., 1986).

### Differential Accumulation of the Secondary Metabolites

**FIGURE 9:**
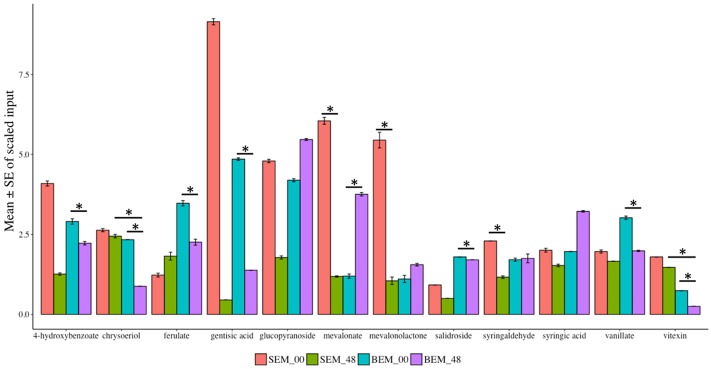
Mean scaled inputs of secondary metabolites and wheat phytochemicals in SEM compared to BEM after 48 h of imbibition compared to 0 h [S-Sukang; B-Baegjoong; and EM-embryo]. Error bars represent ± SE, data was accumulated from three replicates. ^∗^Indicates *p* < 0.05, for more information see Supplementary Table S7.

Various reports have shown that wheat phytochemicals, such as phenolics, tocopherols, carotenoids, and isoflavonoids have crucial antioxidant activity (Zhou et al., 2004). In this study, numerous secondary metabolites (such as alkaloids, benzenoids, flavonoids, and phenylpropanoids) as well as phytochemicals were found to be highly reduced in abundance after 48 h of water-imbibition in PHS-susceptible Baegjoong compared to the PHS-tolerant Sukang when compared to their initial levels at 0 h (**Figure 9** and Supplementary Table S7).

The major secondary metabolites, including vitexin, vanillate, chrysoeriol, 4-hydroxybenzoate, salidroside and gentisic acid, were reduced significantly at the 48 h time point in Baegjoong (**Figure 9**). Previous reports showed that salidroside, which is a phenylethanoid glycoside and was identified in *Rhodiola sachalinensis*, has a potential radical-scavenging function and antioxidant activity at cellular level (Li and Chen, 2001; Yu et al., 2007). We also identified significant differential accumulation of key flavonoids, vitexin, and chrysoeriol, which are also prominent antioxidants. Most importantly, numerous phenylpropanoids (ferulate, syringaldehyde, and vanillate) are more or less constant in Sukang, but reduced in Baegjoong. Considering this observation, it is evident that PHS reduces the phytochemicals along with their antioxidant activity in wheat seeds.

## Conclusion

In general, the baselines for small molecules’ abundance were observed at a much higher level in Sukang, which is a PHS-tolerant wheat cultivar, compared to Baegjoong, which is PHS-susceptible. Baegjoong exhibited signs of increased membrane degradation, which were probably the result of high PLA_2_ abundance at the 48 h time point. Baegjoong also had much higher levels of oxalate at both time points, and the reason for this could be due to a higher lipid degradation (with isocitrate lyase feeding the glyoxylate cycle) and/or from a lower level of oxalate oxidase activity. The precursors of several hormones were found to be differentially regulated between Sukang and Baegjoong at 48 h. For example, in Baegjoong the precursors for IAA were found highly reduced, while the ethylene precursors were found to be highly expressed, which could be responsible for rapid seed germination and eventually it will accelerate the PHS (Keçpczyński and Keçpczyńska, 1997; Matilla, 2000). Although, this metabolic profiling derived information does not reflect the targeted IAA, GA or ethylene levels, it certainly signifies that their precursors or related metabolites have a role to play in response to imbibition. An analysis of the oligosaccharide pathways suggested that Baegjoong contains higher levels of oligosaccharide polymers. Thus, we expect that Baegjoong have a greater amounts of cell wall material, starches, and other polysaccharides, or complex lipids. All of these elements would contribute to the mass, but may precipitate during methanol extraction or for some unknown reasons could not be detected in the analysis (Maharjan and Ferenci, 2003). We also discussed the importance of lipid and oxalate metabolism and how it can affect the seed dormancy. We identified various phospholipids, oxalate and their precursor metabolites in the pathways which are differentially expressed in the two cultivars contrasting for PHS trait. A future study with targeted metabolomics approach can explain if lipid peroxidation occurs during PHS and whether oxalate oxidase can be involved in the production of ROS, which determines the fate of seed dormancy. It is interesting to note that during this study the effects of water-imbibition might be different between the two cultivars. At the time of embryo harvest, while the PHS-susceptible Baegjoong (non-dormant) seeds were at the post-germination stage, the PHS-resistant Sukang (dormant) seeds might be at pre-germination stage. Therefore, this metabolomic profile may also be reflecting physiological stage differences. To distinguish between the metabolites that are specifically the cause of PHS characteristic or the consequences of PHS further ultra-focused experiments are needed. Nonetheless, this study reveals the global scenario of metabolic alterations in two unique wheat cultivars in response to water-imbibition and identifies potential key marker metabolites enriching our understanding of PHS-mechanisms at metabolomic levels.

Current knowledge of metabolomics or chemical biology can be utilized to differentiate the genetic and biochemical mechanisms underlying PHS and seed dormancy (Nonogaki and Nonogaki, 2017). There is a huge scope of applying plant metabolomics toward improving the crop quality and food safety assessment as well as plant metabolic engineering. Plant metabolomics can provide meaningful insight into the numbers of identified metabolites and their associations with each other, and which reflects the agronomic importance of various traits. Thus, metabolomic profile-based knowledge can be used for the generation of more rational models to link specific pathway(s) with quality associated traits (Carreno-Quintero et al., 2013). For example, with this metabolomic profile of imbibed wheat seeds, a more promising study can be performed with the help of the resulting phenotypes with pathway specific metabolites, which will reveal how variation in metabolites abundance/expression can deploy certain phenotypes. One of the potential applications of our metabolomics study would be to generate PHS-resistant varieties by employing metabolomic markers and mQTLs in wheat breeding programs with an overarching aim for improving food security. According to an estimate, the crop yield trends are not encouraging to meet the future food security requirements. It is estimated that the current global wheat yield is increasing at a rate of 0.9% per year, which is far less than the required 2.4% per year to meet projected demands by 2050 (Ray et al., 2013). Pre-harvest sprouted seeds have poor germination at planting, and an inferior crop stand at germination results in comparatively poor yield at the crop harvest. New varieties with better PHS tolerance and with rapid and uniform germination rates at planting will certainly boost wheat yields; and thus will contribute toward improving world’s food security. This metabolic profiling-based finding, especially pathway-based metabolites, can be a guide for the seed dormancy re-programming in wheat to generate PHS-tolerant varieties. Moreover, insights from this metabolic profile can be combined with proteomic and transcriptomic evaluations to gain better understanding of the molecular pathways and enzyme regulations during PHS, which will lead us toward a clearer understanding of various metabolic regulations and cellular mechanisms affecting the PHS.

## Author Contributions

D-WK, RR, and JR designed the experiments. AD, D-WK, and PK performed the experiments. AD, D-WK, PK, RR, and JR analyzed the results and wrote the manuscript.

## Conflict of Interest Statement

The authors declare that the research was conducted in the absence of any commercial or financial relationships that could be construed as a potential conflict of interest.

## Footnotes

1 https://www.R-project.org
