# Supplementary material for: Unraveling Key Metabolomic Alterations in Wheat Embryos Derived from Freshly Harvested and Water-Imbibed Seeds of Two Wheat Cultivars with Contrasting Dormancy Status
Source: Front Plant Sci. 2017 Jul 12;8:1203. doi: 10.3389/fpls.2017.01203 (PMC5506182; doi:10.3389/fpls.2017.01203)
Supplement: Supplementary file 2 [file Table_2.DOCX]

Supplementary Table S2: p-values for various time dependent comparisons between Sukang and Baegjoong for phospholipids.

| **Pathway** | **Metabolites** | **SEM_48 / SEM_00** | **BEM_00 / BEM_48** | **SEM_48 / BEM_48** | **BEM_00 / SEM_00** |
| --- | --- | --- | --- | --- | --- |
|  | *1-linoleoyl-GPE (18:2)* | 0.0758 | 0.1227 | 0.0371 | 0.9264 |
|  | *1-linoleoyl-GPI (18:2)* | 0.5742 | 0.0574 | 0.0211 | 0.0765 |
|  | *1-oleoyl-GPC (18:1)* | 0.2691 | 0.0323 | 0.0582 | 0.5162 |
|  | *1-oleoyl-GPI (18:1)* | 0.4862 | 0.0904 | 0.0379 | 0.3722 |
|  | *1-palmitoyl-GPA (16:0)* | 0.0138 | 0.0352 | 0.0205 | 0.0034 |
|  | *1-palmitoyl-GPE (16:0)* | 0.9464 | 0.0513 | 0.0264 | 0.7145 |
|  | *1-palmitoyl-GPI (16:0)* | 0.0008 | 0.3765 | 0.0596 | 0.6119 |
|  | *1-stearoyl-GPC (18:0)* | 0.8283 | 0.0857 | 0.0447 | 0.8322 |
| **Phospholipid** | *2-palmitoyl-GPC (16:0)* | 0.0151 | 0.1303 | 0.2407 | 1.18E-05 |
|  | *glycerol 3-phosphate* | 0.0292 | 0.1530 | 0.2146 | 0.9332 |
|  | *glycerophosphoethanolamine* | 0.07 | 0.3095 | 0.0219 | 0.8489 |
|  | *glycerophosphorylcholine (GPC)* | 0.7674 | 0.0409 | 0.0893 | 0.5058 |
|  | *1-linolenoyl-GPC (18:3)* | 0.0381 | 0.0614 | 0.0263 | 0.6751 |
|  | *1-palmitoyl-GPG (16:0)* | 0.2927 | 0.0793 | 0.0289 | 0.0268 |
|  | *1-oleoyl-GPA (18:1)* | 0.0015 | 0.0323 | 0.0582 | 0.0635 |
|  | *glycerophosphoinositol* | 0.0284 | 0.0665 | 0.3128 | 0.1744 |
|  | *1-palmitoyl-2-linoleoyl-GPC (16:0/18:2)* | 0.1111 | 0.0492 | 0.0144 | 0.025 |
|  | *1-palmitoyl-2-oleoyl-GPC (16:0/18:1)* | 0.0476 | 0.0728 | 0.3138 | 0.0012 |
