# Supplementary material for: Unraveling Key Metabolomic Alterations in Wheat Embryos Derived from Freshly Harvested and Water-Imbibed Seeds of Two Wheat Cultivars with Contrasting Dormancy Status
Source: Front Plant Sci. 2017 Jul 12;8:1203. doi: 10.3389/fpls.2017.01203 (PMC5506182; doi:10.3389/fpls.2017.01203)
Supplement: Supplementary file 3 [file Table_3.DOCX]

Supplementary Table S3: p-values for various time dependent comparisons between Sukang and Baegjoong for carbohydrate, auxin and ethylene metabolism.

| **Pathway** | **Metabolites** | **SEM_00 / SEM_48** | **BEM_00 / BEM_48** | **SEM_48 / BEM_48** | | **SEM_00 / BEM_00** | |
| --- | --- | --- | --- | --- | --- | --- | --- |
|  | Oxalate | 0.0001 | 1.52E-09 | 1.20E-08 | 0.3551 | |  |
| Carbohydrates | Isocitrate | 2.25E-11 | 4.54E-15 | 2.20E-16 | 2.62E-02 | |  |
|  | Dehydroascorbate | 4.92E-09 | 2.20E-16 | 2.20E-16 | 8.29E-01 | |  |
|  | Indoleacetylaspartate | 2.38E-07 | 2.20E-16 | 2.20E-16 | 8.40E-05 | |  |
|  | Indolelactate | 8.32E-10 | 2.35E-11 | 0.0006 | 1.20E-08 | |  |
| Auxin-related | Tryptophan | 0.7073 | 7.13E-12 | 5.78E-13 | 2.00E-10 | |  |
| compounds | Indole-3-carboxylic acid | 4.09E-05 | 0.2027 | 0.1241 | 8.13E-05 | |  |
|  | 2-oxindole-3-acetate | 8.05E-13 | 2.20E-16 | 2.20E-16 | 3.25E-07 | |  |
|  | Serotonin | 0.7615 | 5.51E-14 | 2.59E-12 | 0.2980 | |  |
|  | Cyano-alanine | 7.23E-06 | 1.62E-15 | 6.11E-16 | 1.99E-07 | |  |
| Ethylene | Methionine | 5.78E-12 | 2.20E-16 | 2.20E-16 | 5.38E-06 | |  |
| precursors | SAM | 1.10E-09 | 1.41E-05 | 9.21E-10 | 0.2670 | |  |
