# Supplementary material for: Unraveling Key Metabolomic Alterations in Wheat Embryos Derived from Freshly Harvested and Water-Imbibed Seeds of Two Wheat Cultivars with Contrasting Dormancy Status
Source: Front Plant Sci. 2017 Jul 12;8:1203. doi: 10.3389/fpls.2017.01203 (PMC5506182; doi:10.3389/fpls.2017.01203)
Supplement: Supplementary file 4 [file Table_4.DOCX]

Supplementary Table S4: p-values for various time dependent comparisons between Sukang and Baegjoong for raffinose family oligosaccharides (RFOs).

| **Metabolites** | **SEM_48 / SEM_00** | **BEM_00 / BEM_48** | **SEM_48 / BEM_48** | **BEM_00 / SEM_00** |
| --- | --- | --- | --- | --- |
| *glucose* | 0.0049 | 0.0340 | 0.0921 | 0.1845 |
| *fructose 1,6-diphosphate* | 0.0118 | 0.2344 | 0.0266 | 0.1183 |
| *gluconate* | 5.53E-06 | 0.0565 | 0.2018 | 0.2937 |
| *fructose* | 0.0554 | 0.1820 | 0.4425 | 0.8111 |
| *galactinol* | 0.3793 | 0.0731 | 0.0449 | 0.3578 |
| *galactitol (dulcitol)* | 0.0294 | 0.4575 | 0.1779 | 0.0704 |
| *galactonate* | 0.0031 | 0.0233 | 0.2534 | 0.6782 |
| *galactose* | 0.1091 | 0.3666 | 0.0300 | 0.0690 |
| *maltose* | 0.0007 | 0.0046 | 0.0224 | 0.0093 |
| *mannitol* | 0.0493 | 0.0082 | 0.0802 | 0.3053 |
| *mannose* | 0.0026 | 0.0476 | 0.4005 | 0.0997 |
| *raffinose* | 0.2069 | 0.0013 | 0.0021 | 0.6241 |
| *sorbitol* | 2.66E-07 | 0.0168 | 0.0906 | 0.8387 |
| *sucrose* | 0.7906 | 0.3891 | 0.0021 | 0.3414 |
| *verbascose* | 0.0432 | 0.1938 | 0.0264 | 0.1501 |
| *myo-inositol* | 0.0209 | 0.0155 | 0.0926 | 0.3198 |
