# Supplementary material for: Unraveling Key Metabolomic Alterations in Wheat Embryos Derived from Freshly Harvested and Water-Imbibed Seeds of Two Wheat Cultivars with Contrasting Dormancy Status
Source: Front Plant Sci. 2017 Jul 12;8:1203. doi: 10.3389/fpls.2017.01203 (PMC5506182; doi:10.3389/fpls.2017.01203)
Supplement: Supplementary file 5 [file Table_5.DOCX]

**Table S5**: Differential accumulation levels of various amino acids in Baegjoong and Sukang as detected by LC-MS and GC-MS. The serial numbers of metabolites in this table were used for constructing the Fig. S7.

Differential expression of various amino acid in Baegjoong (B) and Sukang (S) detected by LC-MS and GC-MS.

Number 1-13: Serine family phosphoglycerate derived amino acids;

Number 14-37: Aromatic amino acid metabolism (PEP derived);

Number 38-67: Aspartate family (OAA derived);

Number 68-102: Glutamate family (alpha-keto-glutarate derived);

Number 103-104: Branched chain amino acids (OAA derived);

Number 105-121: Branched chain amino acids (Pyruvate derived);

Number 122-128: amines and polyamines;

Number 129-137: Amino acid involved in glutathione metabolism

| **Amino acid** | | **Mean values (scaled input data)** | | | | |
| --- | --- | --- | --- | --- | --- | --- |
| **Serial No.** | **Biochemical Name** | **SEM_00** | **SEM_48** | **BEM_00** | **BEM_48** |  |
| **1** | **Betaine** | 1.55 | 1.23 | 1.55 | 1.90 |  |
| **2** | **Cysteine** | 1.03 | 1.97 | 1.15 | 5.69 |  |
| **3** | **Cystine** | 0.16 | 0.51 | 0.22 | 0.47 |  |
| **4** | **Dimethylglycine** | 1.98 | 1.34 | 2.17 | 13.27 |  |
| **5** | **Glycine** | 2.02 | 2.09 | 2.14 | 3.65 |  |
| **6** | **Hypotaurine** | 0.83 | 0.28 | 1.59 | 0.28 |  |
| **7** | **N-acetylserine** | 1.18 | 1.72 | 0.47 | 5.03 |  |
| **8** | **O-acetylserine** | 0.50 | 1.43 | 0.38 | 14.06 |  |
| **9** | **Serine** | 1.04 | 4.03 | 1.94 | 10.71 |  |
| **10** | **Taurine** | 1.12 | 1.21 | 2.48 | 1.32 |  |
| **11** | **S-methylcysteine** | 0.54 | 1.10 | 0.88 | 1.71 |  |
| **12** | **N-acetyltaurine** | 0.81 | 0.81 | 1.19 | 8.20 |  |
| **13** | **Sulfate*** | 1.08 | 1.17 | 1.73 | 2.14 |  |
| **14** | **3-(4-hydroxyphenyl) lactate** | 1.23 | 1.34 | 0.82 | 0.38 |  |
| **15** | **3-methoxytyrosine** | 0.74 | 1.27 | 1.57 | 17.58 |  |
| **16** | **4-hydroxyphenylpyruvate** | 0.78 | 1.10 | 0.78 | 4.07 |  |
| **17** | **Indolelactate** | 0.60 | 0.27 | 0.97 | 0.19 |  |
| **18** | **Kynurenate** | 1.40 | 0.23 | 1.80 | 0.47 |  |
| **19** | **Kynurenine** | 0.52 | 0.48 | 0.91 | 1.23 |  |
| **20** | **N-acetylphenylalanine** | 2.26 | 0.34 | 1.99 | 1.37 |  |
| **21** | **N-acetyltryptophan** | 0.74 | 0.25 | 1.46 | 1.07 |  |
| **22** | **N-acetyltyrosine** | 1.01 | 0.54 | 0.73 | 2.01 |  |
| **23** | **Phenethylamine** | 0.61 | 0.87 | 0.72 | 6.33 |  |
| **24** | **Phenylacetate** | 1.50 | 0.56 | 1.38 | 1.80 |  |
| **25** | **Phenylalanine** | 0.93 | 2.42 | 1.30 | 11.08 |  |
| **26** | **Phenyllactate (PLA)** | 1.85 | 1.60 | 1.23 | 1.16 |  |
| **27** | **Phenylpyruvate** | 0.26 | 0.85 | 0.14 | 4.09 |  |
| **28** | **Quinate** | 1.92 | 1.22 | 0.39 | 9.00 |  |
| **29** | **Serotonin** | 0.06 | 0.06 | 0.06 | 1.44 |  |
| **30** | **Shikimate** | 1.68 | 2.22 | 0.51 | 26.19 |  |
| **31** | **Tryptophan** | 0.51 | 0.49 | 1.02 | 2.01 |  |
| **32** | **Tyrosine** | 0.94 | 2.34 | 1.29 | 8.90 |  |
| **33** | **3-(4-hydroxyphenyl) propionate** | 1.22 | 1.14 | 1.34 | 8.20 |  |
| **34** | **O-sulfo-L-tyrosine** | 0.81 | 0.90 | 0.86 | 0.85 |  |
| **35** | **Tryptamine** | 0.03 | 0.03 | 0.63 | 9.27 |  |
| **36** | **Tyramine** | 0.69 | 0.69 | 0.69 | 0.95 |  |
| **37** | **N-formylphenylalanine** | 1.17 | 1.22 | 1.15 | 4.99 |  |
| **38** | **2-aminoadipate** | 1.45 | 1.07 | 0.56 | 3.22 |  |
| **39** | **Alanine** | 1.25 | 5.00 | 1.72 | 4.74 |  |
| **40** | **Asparagine** | 2.26 | 1.53 | 3.15 | 2.81 |  |
| **41** | **Aspartate** | 1.76 | 0.72 | 1.19 | 4.05 |  |
| **42** | **Homoserine** | 1.31 | 2.26 | 1.18 | 11.50 |  |
| **43** | **Lysine** | 0.96 | 2.62 | 1.81 | 4.12 |  |
| **44** | **Methionine** | 1.02 | 2.77 | 1.25 | 10.32 |  |
| **45** | **Methionine sulfoxide** | 1.02 | 1.43 | 1.08 | 3.02 |  |
| **46** | **N2-acetyllysine** | 2.15 | 2.18 | 0.40 | 0.46 |  |
| **47** | **N6-acetyllysine** | 1.40 | 7.42 | 1.89 | 12.46 |  |
| **48** | **N-6-trimethyllysine** | 1.81 | 2.41 | 1.96 | 1.71 |  |
| **49** | **N-acetylalanine** | 1.13 | 2.35 | 1.20 | 2.41 |  |
| **50** | **N-acetylasparagine** | 4.95 | 1.03 | 4.30 | 4.49 |  |
| **51** | **N-acetylaspartate (NAA)** | 1.08 | 0.08 | 0.89 | 3.25 |  |
| **52** | **N-acetyl-beta-alanine** | 1.63 | 1.84 | 0.77 | 2.63 |  |
| **53** | **N-acetylmethionine** | 1.63 | 2.02 | 1.88 | 2.65 |  |
| **54** | **N-acetylthreonine** | 1.54 | 2.95 | 0.64 | 3.16 |  |
| **55** | **N-formylmethionine** | 0.24 | 2.33 | 0.22 | 4.74 |  |
| **56** | **Pipecolate** | 3.20 | 1.90 | 2.64 | 4.56 |  |
| **57** | **Saccharopine** | 0.27 | 1.18 | 0.24 | 0.24 |  |
| **58** | **S-adenosylhomocysteine (SAH)** | 0.90 | 1.60 | 0.92 | 1.10 |  |
| **59** | **S-adenosylmethionine (SAM)** | 0.93 | 0.93 | 0.93 | 0.99 |  |
| **60** | **Threonine** | 1.05 | 2.75 | 1.43 | 5.92 |  |
| **61** | **6-oxopiperidine-2-carboxylic acid** | 3.14 | 0.43 | 1.09 | 1.45 |  |
| **62** | **2-piperidinone** | 1.18 | 1.02 | 1.09 | 0.81 |  |
| **63** | **N-acetylmethionine sulfoxide** | 4.52 | 1.07 | 2.76 | 2.10 |  |
| **64** | **2-hydroxyadipate** | 1.20 | 1.16 | 0.44 | 1.30 |  |
| **65** | **N-methylpipecolate** | 1.43 | 1.45 | 1.72 | 2.47 |  |
| **66** | **S-methylmethionine** | 1.24 | 23.93 | 0.30 | 90.31 |  |
| **67** | **Methionine sulfone** | 0.85 | 1.24 | 0.25 | 1.67 |  |
| **68** | **1,3-diaminopropane** | 0.50 | 0.91 | 1.26 | 5.00 |  |
| **69** | **2-aminobutyrate** | 3.00 | 1.04 | 2.60 | 3.16 |  |
| **70** | **2-pyrrolidinone** | 8.69 | 1.52 | 7.12 | 0.95 |  |
| **71** | **3-methylhistidine** | 0.34 | 1.13 | 0.34 | 7.18 |  |
| **72** | **4-acetamidobutanoate** | 1.91 | 1.24 | 1.08 | 1.55 |  |
| **73** | **4-hydroxybutyrate (GHB)** | 1.45 | 0.60 | 1.07 | 1.12 |  |
| **74** | **Arginine** | 1.10 | 3.00 | 3.75 | 6.34 |  |
| **75** | **Argininosuccinate** | 1.14 | 6.13 | 0.40 | 0.81 |  |
| **76** | **Carboxyethyl-GABA** | 1.40 | 0.65 | 1.00 | 0.91 |  |
| **77** | **Citrulline** | 0.30 | 3.11 | 1.11 | 1.23 |  |
| **78** | **Dimethylarginine (SDMA + ADMA)** | 1.62 | 2.53 | 3.39 | 4.19 |  |
| **79** | **Ergothioneine** | 5.55 | 0.91 | 6.43 | 0.34 |  |
| **80** | **Gamma-aminobutyrate (GABA)** | 1.38 | 3.63 | 1.27 | 10.89 |  |
| **81** | **Glutamate** | 1.72 | 2.35 | 1.40 | 1.89 |  |
| **82** | **Glutamate, gamma-methyl ester** | 2.33 | 1.43 | 1.88 | 1.95 |  |
| **83** | **Glutamine** | 0.72 | 5.66 | 1.09 | 47.75 |  |
| **84** | **Histamine** | 0.15 | 1.77 | 1.78 | 11.32 |  |
| **85** | **Histidine** | 1.64 | 4.18 | 2.91 | 19.39 |  |
| **86** | **Homocitrulline** | 0.54 | 1.09 | 1.48 | 1.73 |  |
| **87** | **N-acetylarginine** | 0.81 | 0.90 | 0.98 | 6.74 |  |
| **88** | **N-acetylglutamate** | 1.34 | 1.80 | 0.73 | 8.96 |  |
| **89** | **N-acetylglutamine** | 4.05 | 0.78 | 1.10 | 12.42 |  |
| **90** | **N-alpha-acetylornithine** | 0.69 | 1.64 | 0.23 | 1.05 |  |
| **91** | **N-acetylproline** | 0.95 | 0.76 | 1.39 | 2.42 |  |
| **92** | **Ornithine** | 0.79 | 2.09 | 1.46 | 2.00 |  |
| **93** | **Proline** | 0.71 | 4.95 | 1.92 | 10.97 |  |
| **94** | **Pyroglutamine*** | 1.47 | 1.20 | 1.15 | 2.47 |  |
| **95** | **Stachydrine** | 1.03 | 1.05 | 1.40 | 1.66 |  |
| **96** | **Trans-4-hydroxyproline** | 1.09 | 1.06 | 0.73 | 1.66 |  |
| **97** | **N-methylproline** | 1.36 | 1.14 | 1.52 | 2.23 |  |
| **98** | **4-hydroxy-2-oxoglutaric acid** | 2.06 | 0.14 | 1.13 | 2.21 |  |
| **99** | **N-delta-acetylornithine** | 1.16 | 2.15 | 1.01 | 3.20 |  |
| **100** | **N-monomethylarginine** | 0.64 | 1.08 | 1.42 | 1.63 |  |
| **101** | **4-guanidinobutanoate** | 2.31 | 2.00 | 1.63 | 1.15 |  |
| **102** | **4-imidazoleacetate** | 0.83 | 0.58 | 1.16 | 1.91 |  |
| **103** | **Isoleucine** | 1.01 | 2.70 | 1.26 | 18.01 |  |
| **104** | **Allo-isoleucine** | 1.93 | 1.33 | 1.77 | 1.95 |  |
| **105** | **2,3-dihydroxyisovalerate** | 12.13 | 1.20 | 1.12 | 10.30 |  |
| **106** | **2-hydroxy-3-methylvalerate** | 3.64 | 1.25 | 1.56 | 0.93 |  |
| **107** | **3-hydroxyisobutyrate** | 1.09 | 0.57 | 0.57 | 0.57 |  |
| **108** | **3-methyl-2-oxobutyrate** | 2.18 | 1.03 | 0.66 | 1.91 |  |
| **109** | **3-methyl-2-oxovalerate** | 2.75 | 2.60 | 2.04 | 14.81 |  |
| **110** | **4-methyl-2-oxopentanoate** | 2.36 | 2.31 | 1.98 | 12.40 |  |
| **111** | **Alpha-hydroxyisocaproate** | 1.74 | 2.46 | 0.96 | 1.82 |  |
| **112** | **Alpha-hydroxyisovalerate** | 4.16 | 1.10 | 1.75 | 0.88 |  |
| **113** | **Beta-hydroxyisovalerate** | 0.91 | 0.66 | 1.13 | 1.86 |  |
| **114** | **Leucine** | 0.89 | 2.54 | 1.18 | 14.14 |  |
| **115** | **Methylsuccinate** | 2.10 | 1.46 | 2.05 | 2.94 |  |
| **116** | **N-acetylisoleucine** | 1.40 | 0.38 | 1.09 | 2.25 |  |
| **117** | **N-acetylleucine** | 1.27 | 0.24 | 1.04 | 1.25 |  |
| **118** | **N-acetylvaline** | 2.77 | 0.41 | 2.03 | 1.13 |  |
| **119** | **Valine** | 1.59 | 2.55 | 1.87 | 9.61 |  |
| **120** | **2-isopropylmalate** | 10.11 | 0.15 | 2.47 | 4.59 |  |
| **121** | **Isovalerylcarnitine** | 1.00 | 1.00 | 1.00 | 1.00 |  |
| **122** | **5-methylthioadenosine (MTA)** | 0.08 | 0.51 | 0.08 | 9.66 |  |
| **123** | **Agmatine** | 1.34 | 2.42 | 3.27 | 20.45 |  |
| **124** | **Putrescine** | 0.99 | 3.60 | 2.18 | 30.23 |  |
| **125** | **Spermidine** | 0.88 | 0.70 | 1.89 | 14.76 |  |
| **126** | **Feruloylputrescine** | 0.17 | 0.17 | 0.33 | 1.93 |  |
| **127** | **N-acetylputrescine** | 0.75 | 5.51 | 0.95 | 5.26 |  |
| **128** | **Nicotianamine** | 0.75 | 0.58 | 0.58 | 5.49 |  |
| **129** | **5-oxoproline** | 4.17 | 1.07 | 4.30 | 3.69 |  |
| **130** | **Cysteine-glutathione disulfide** | 0.63 | 2.97 | 1.36 | 7.58 |  |
| **131** | **Gamma-glutamyl-2-aminobutyrate** | 0.83 | 0.81 | 1.35 | 0.81 |  |
| **132** | **Glutathione, oxidized (GSSG)** | 1.05 | 3.97 | 1.72 | 8.90 |  |
| **133** | **Glutathione, reduced (GSH)** | 9.65 | 0.82 | 7.78 | 0.82 |  |
| **134** | **Ophthalmate** | 0.43 | 1.64 | 1.01 | 4.98 |  |
| **135** | **S-methylglutathione** | 0.84 | 1.20 | 1.01 | 2.60 |  |
| **136** | **Norophthalmate*** | 0.74 | 2.90 | 2.48 | 5.11 |  |
| **137** | **4-hydroxy-nonenal-glutathione** | 0.61 | 0.43 | 1.41 | 3.25 |  |
