# Supplementary material for: Unraveling Key Metabolomic Alterations in Wheat Embryos Derived from Freshly Harvested and Water-Imbibed Seeds of Two Wheat Cultivars with Contrasting Dormancy Status
Source: Front Plant Sci. 2017 Jul 12;8:1203. doi: 10.3389/fpls.2017.01203 (PMC5506182; doi:10.3389/fpls.2017.01203)
Supplement: Supplementary file 6 [file Table_6.DOCX]

Supplementary Table S6: p-values for various time dependent comparisons between Sukang and Baegjoong for aromatic amino acids.

| **Pathway** | **Metabolites** | **SEM_00 / SEM_48** | **BEM_00 / BEM_48** | **SEM_48 / BEM_48** | **SEM_00 / BEM_00** |
| --- | --- | --- | --- | --- | --- |
|  | *3-methoxytyrosine* | 0.0428 | 0.0458 | 0.0248 | 0.0073 |
|  | *4-hydroxyphenylpyruvate* | 0.0642 | 0.1252 | 0.4481 | 0.9172 |
|  | *N-acetylphenylalanine* | 6.51E-05 | 0.1355 | 0.2113 | 0.6724 |
|  | *N-acetyltryptophan* | 0.001 | 0.1071 | 0.1523 | 0.0198 |
|  | *N-acetyltyrosine* | 0.0002 | 0.0740 | 0.1136 | 0.0196 |
| **Aromatic** | *phenethylamine* | 0.0039 | 0.0846 | 0.0880 | 0.1218 |
| **Amino** | *phenylacetate* | 0.0095 | 0.2113 | 2.20E-15 | 0.4552 |
| **acid** | *phenylalanine* | 0.0004 | 0.0200 | 0.1032 | 0.1412 |
|  | *phenyllactate (PLA)* | 0.2675 | 0.0573 | 0.0004 | 0.0087 |
|  | *phenylpyruvate* | 2.58E-05 | 2.20E-15 | 2.20E-15 | 0.1011 |
|  | *quinate* | 0.0434 | 0.1909 | 0.0195 | 5.66E-07 |
|  | *shikimate* | 0.1691 | 0.1999 | 0.0021 | 8.74E-06 |
|  | *tyrosine* | 7.71E-05 | 0.0148 | 0.1345 | 0.0855 |
|  | *N-formylphenylalanine* | 0.9183 | 0.0180 | 0.0280 | 0.9275 |
