# Supplementary material for: Unraveling Key Metabolomic Alterations in Wheat Embryos Derived from Freshly Harvested and Water-Imbibed Seeds of Two Wheat Cultivars with Contrasting Dormancy Status
Source: Front Plant Sci. 2017 Jul 12;8:1203. doi: 10.3389/fpls.2017.01203 (PMC5506182; doi:10.3389/fpls.2017.01203)
Supplement: Supplementary file 7 [file Table_7.DOCX]

Supplementary Table S7: p-values for various time dependent comparisons between Sukang and Baegjoong for secondary metabolites and phytochemicals.

| **Pathway** | **Metabolites** | **SEM_00 / SEM_48** | **BEM_00 / BEM_48** | **SEM_48 / BEM_48** | **SEM_00 / BEM_00** |
| --- | --- | --- | --- | --- | --- |
| Alkaloids | *salidroside* | 4.87E-09 | 2E-07 | 2E-07 | 1.45E-09 |
|  | *2,4,6-trihydroxybenzoate* | 0.2085 | 2.2E-07 | 0.0693 | 0.1308 |
| Benzenoids | *4-hydroxybenzoate* | 3.93E-06 | 0.0320 | 0.3005 | 0.0618 |
|  | *hydroquinone beta-D-glucopyranoside* | 0.0093 | 0.0413 | 0.0078 | 0.6967 |
| Flavonoids | *vitexin* | 0.0491 | 2E-07 | 2E-07 | 1.89E-07 |
|  | *chrysoeriol* | 0.6823 | 2E-07 | 0.0177 | 0.5074 |
|  | *ferulate* | 0.1944 | 0.1761 | 0.0314 | 0.0016 |
| Phenyl- | *syringaldehyde* | 0.0049 | 0.0433 | 0.4017 | 0.1829 |
| propanoids | *syringic acid* | 0.1788 | 0.0345 | 0.0072 | 0.9126 |
|  | *vanillate* | 0.2326 | 0.0279 | 0.0160 | 0.0077 |
|  | *gentisic acid-5-glucoside* | 2.30E-10 | 0.0233 | 2E-07 | 0.0112 |
| Terpenoids | *mevalonate* | 2.54E-06 | 0.0254 | 0.0636 | 2.54E-06 |
|  | *mevalonolactone* | 0.0002 | 0.0618 | 0.1984 | 0.0002 |
