# Supplementary material for: Unraveling Key Metabolomic Alterations in Wheat Embryos Derived from Freshly Harvested and Water-Imbibed Seeds of Two Wheat Cultivars with Contrasting Dormancy Status
Source: Front Plant Sci. 2017 Jul 12;8:1203. doi: 10.3389/fpls.2017.01203 (PMC5506182; doi:10.3389/fpls.2017.01203)
Supplement: Supplementary file 8 [file Data_Sheet_1.DOCX]

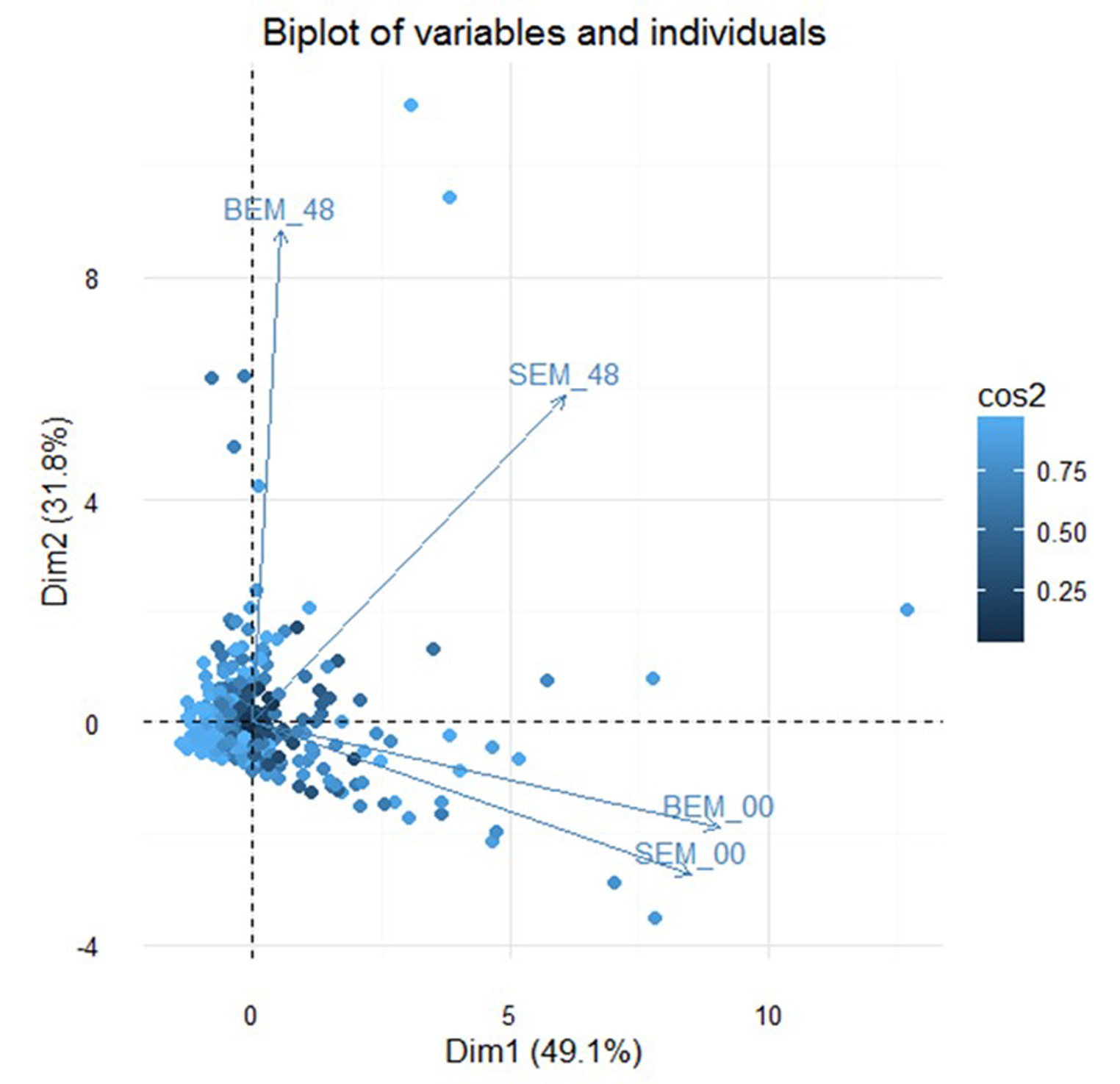


**Figure S1**: PCA of all the metabolites that were identified in this study using R 3.3.1 software. B: Baegjoong; S: Sukang; EM: embryo; Time points: 0 h and 48 h. PCA of log-scaled putative metabolites originating from two cultivars, i.e., Sukang and Baegjoong. All the vectors in the PCA span an n-dimensional space provide the finest sample separation where each point represents a linear combination of all of the metabolites from either Sukang or Baegjoong [cos2 = the quality of the individuals on the factor map].

**Figure S2**: Overall metabolic comparison of Sukang and Baegjoong at two time points. Y axis represents mean value ± SE of 409 metabolites while x-axis shows treatments. p-value of each cultivar with 0 h versus 48 h is also specified in the figure.


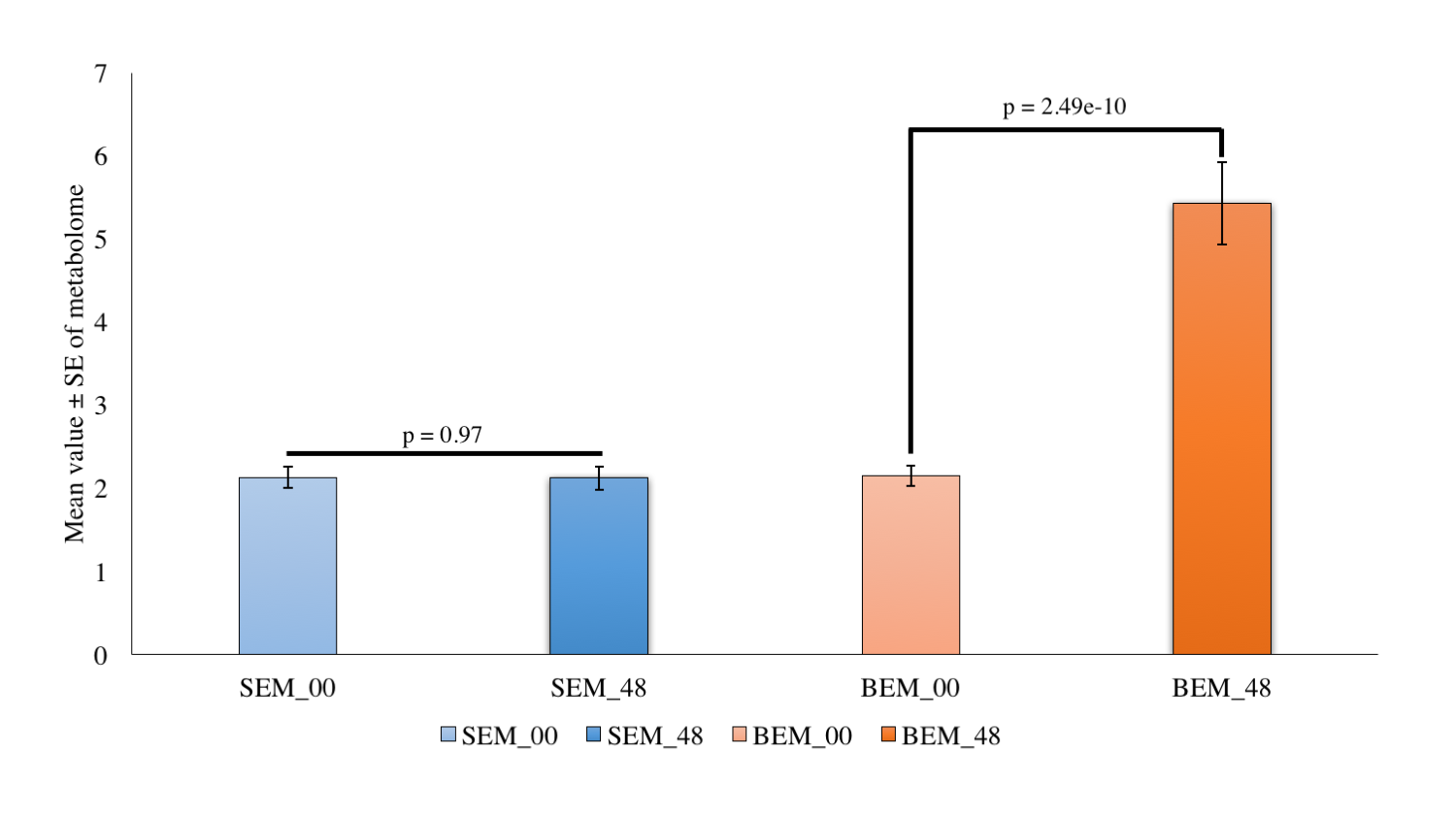

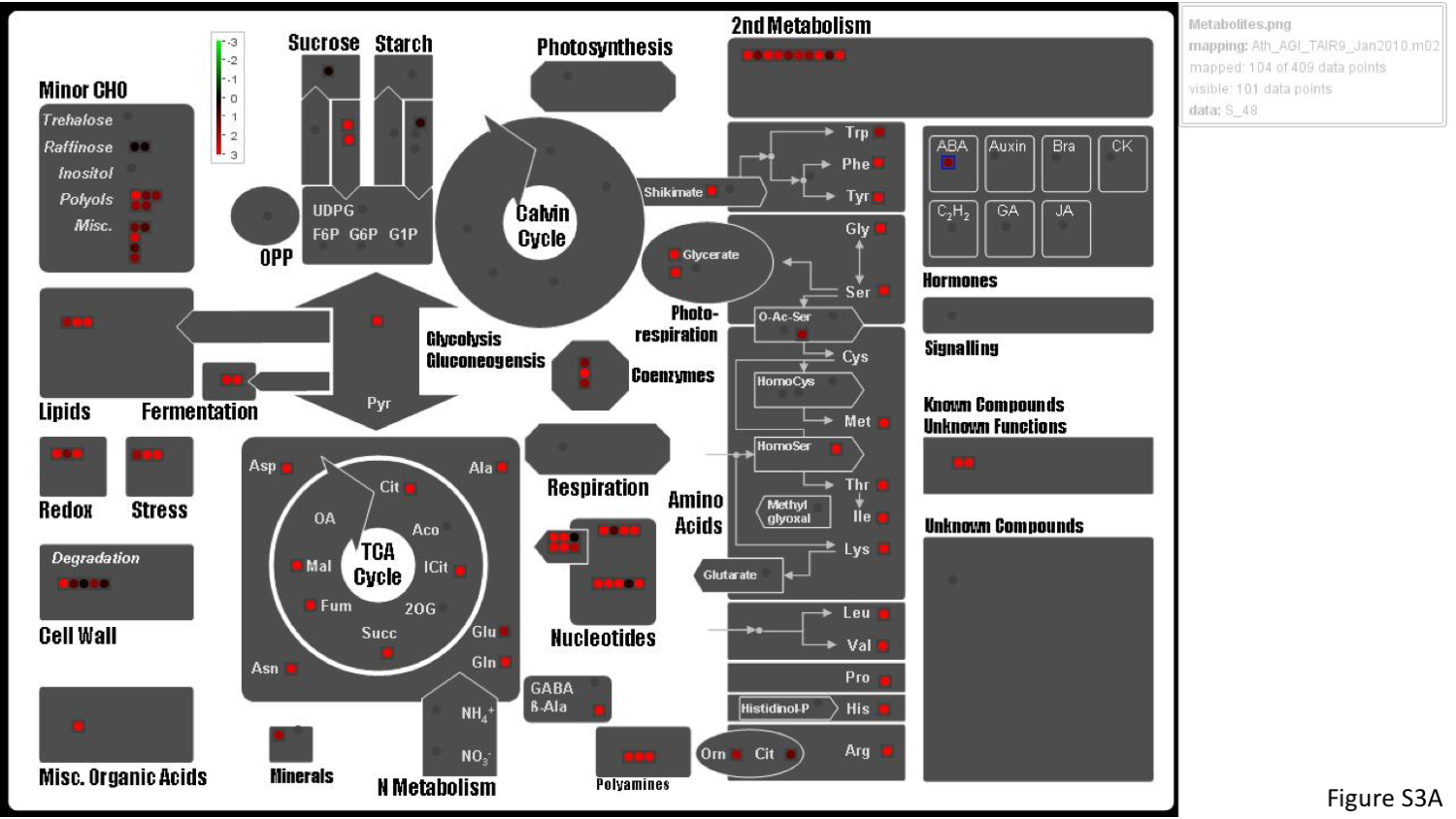


**Figure S3 (A)**: Mapping of identified embryonic metabolites on MapMan. (A) One hundred one data points (colored dots) on MapMan were mapped as metabolites of interest for BEM_48 h samples. Color and its intensity shows the abundance levels of a particular metabolite.


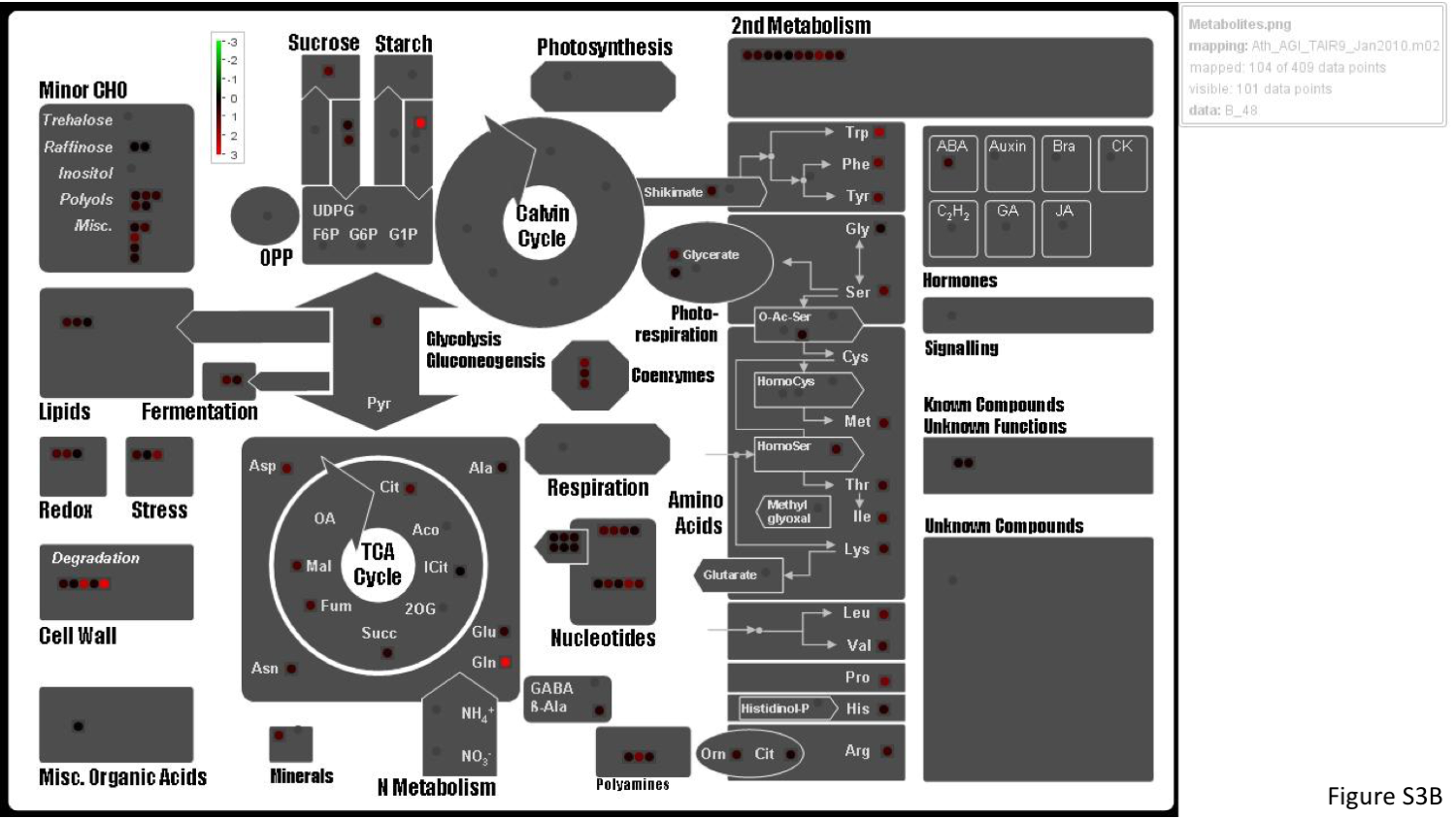


**Figure S3 (B)**: Mapping of identified embryonic metabolites on MapMan. (B) One hundred one data points (colored dots) on MapMan were mapped as metabolites of interest for SEM_48 h samples. Color and its intensity shows the abundance levels of particular metabolites.


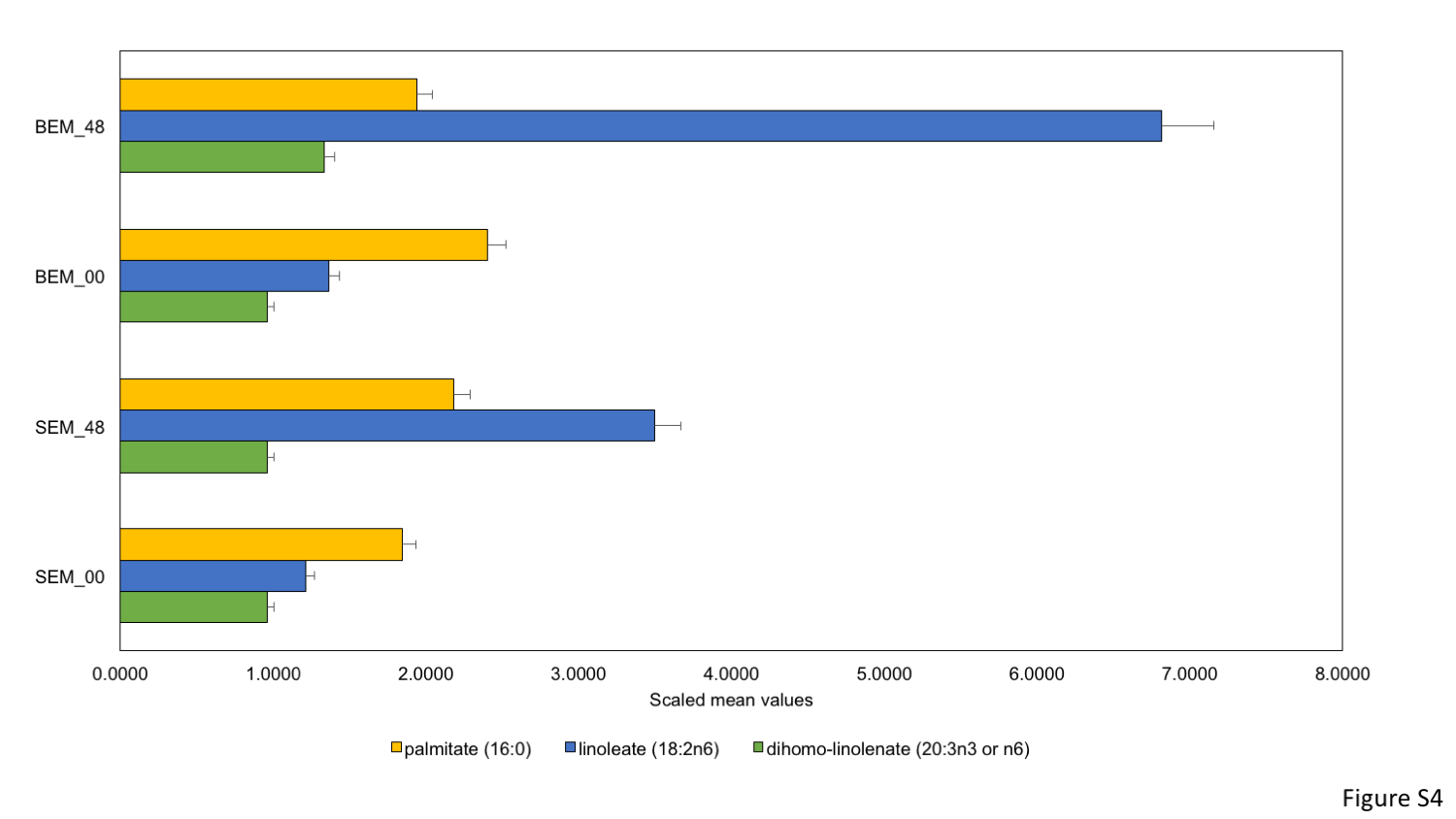


**Figure S4**: Increased levels of free fatty acid such as linoleate, linolenate and palmitate, suggested that the reversal effect of pre-mature germination is mediated by the increased PLA_2_ activity, which is steadily detected in Baegjoong. The X axis shows metabolites’ abundance, and Y axis shows sample types.


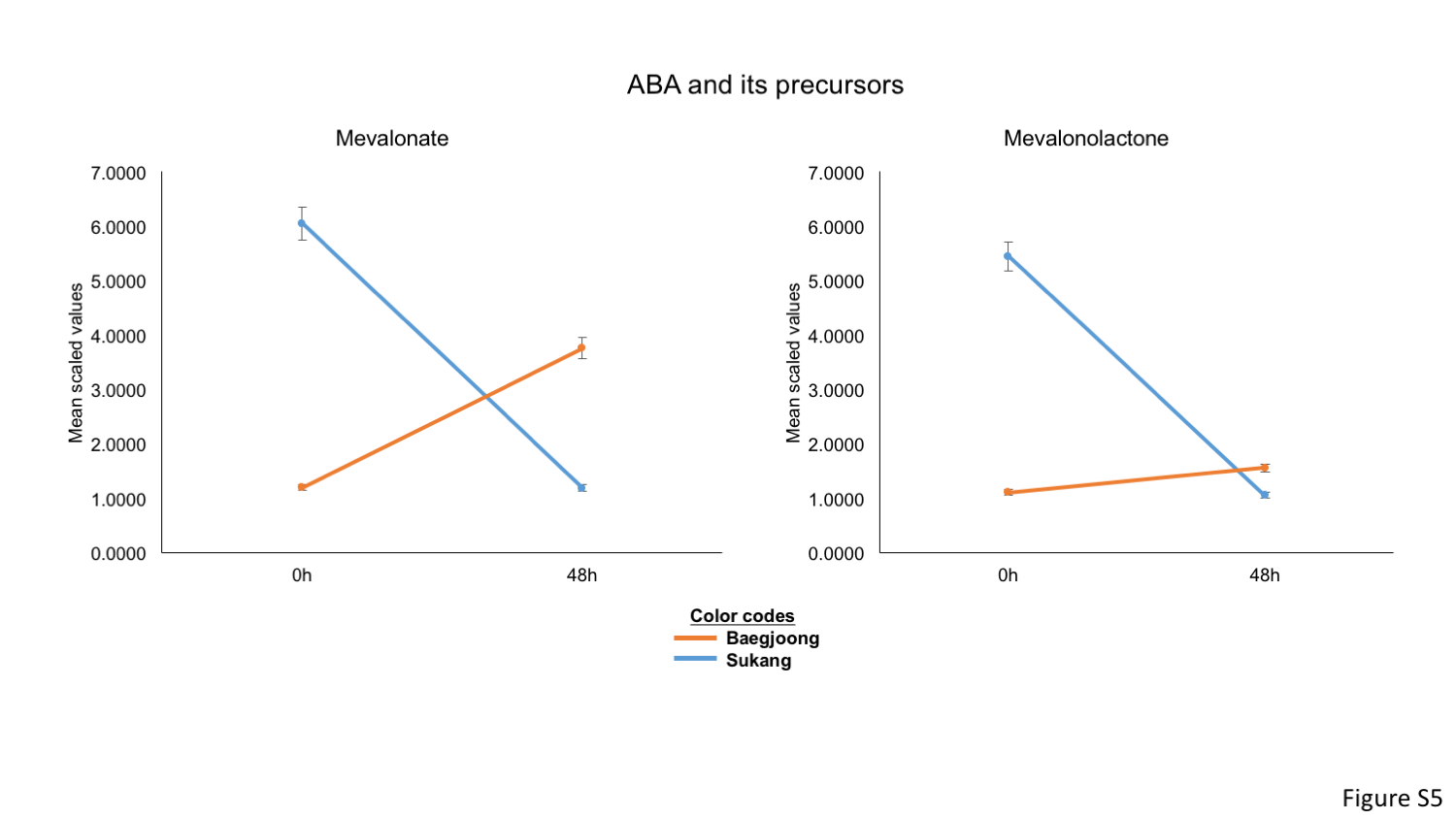


**Figure S5**: Enhanced accumulation of mevalonate and mevalonolactone was observed in Baegjoong after 48 h of imbibition. Right panel is for mevalonolactone, and the left panel is for mevalonate. In both panel X axis shows the time point of tissue harvest, and Y axis shows metabolites’ abundance. Baegjoong data is represented by orange line, and Sukang data is represented by blue line.


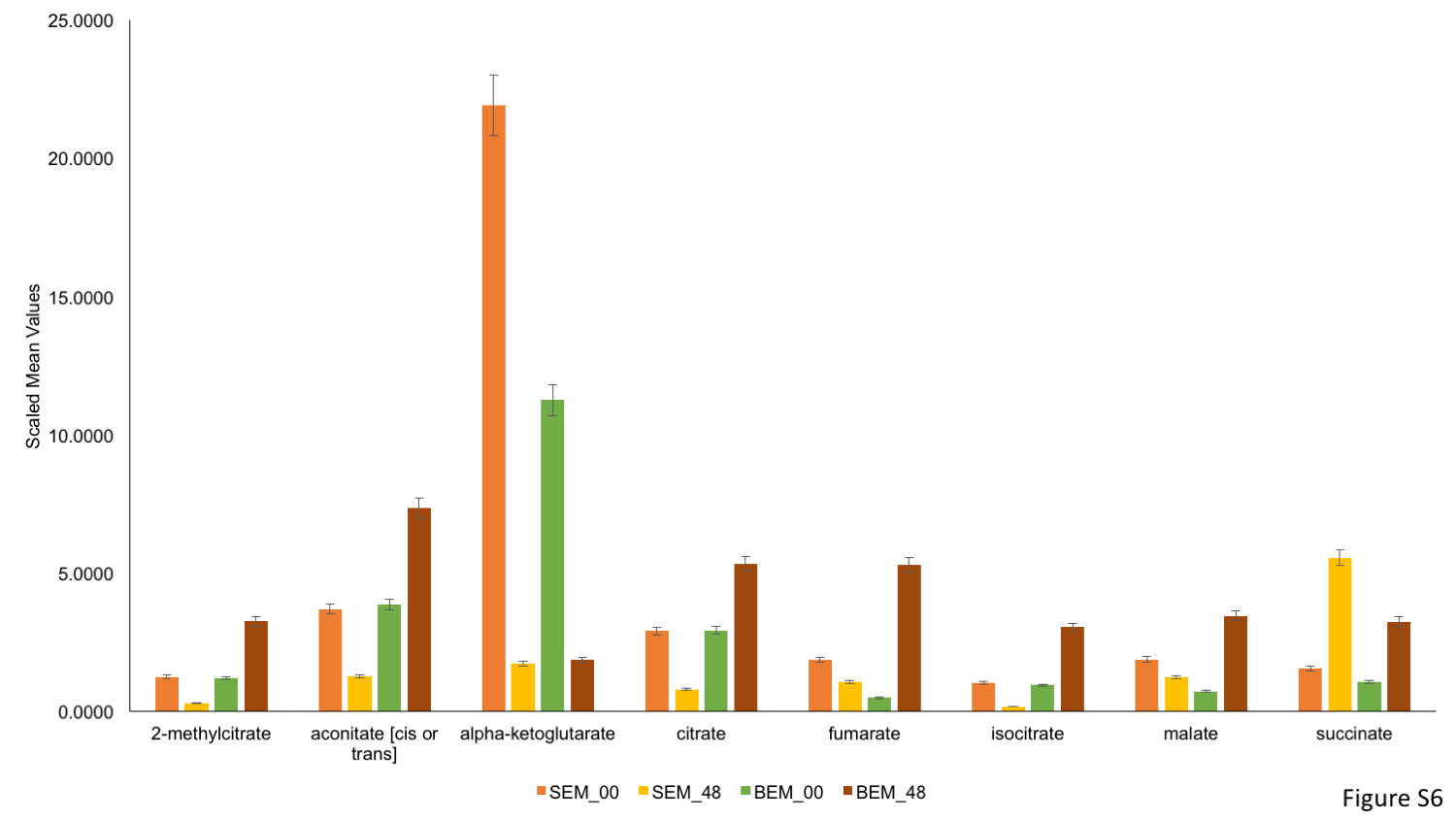


**Figure S6**: Metabolites of TCA cycle such as: cis-aconitate, citrate, fumarate, isocitrate, malate, and succinate were significantly increased in Baegjoong compared to Sukang after 48 h of imbibition. X axis shows different metabolites, and Y axis shows metabolites’ abundance in the embryos.


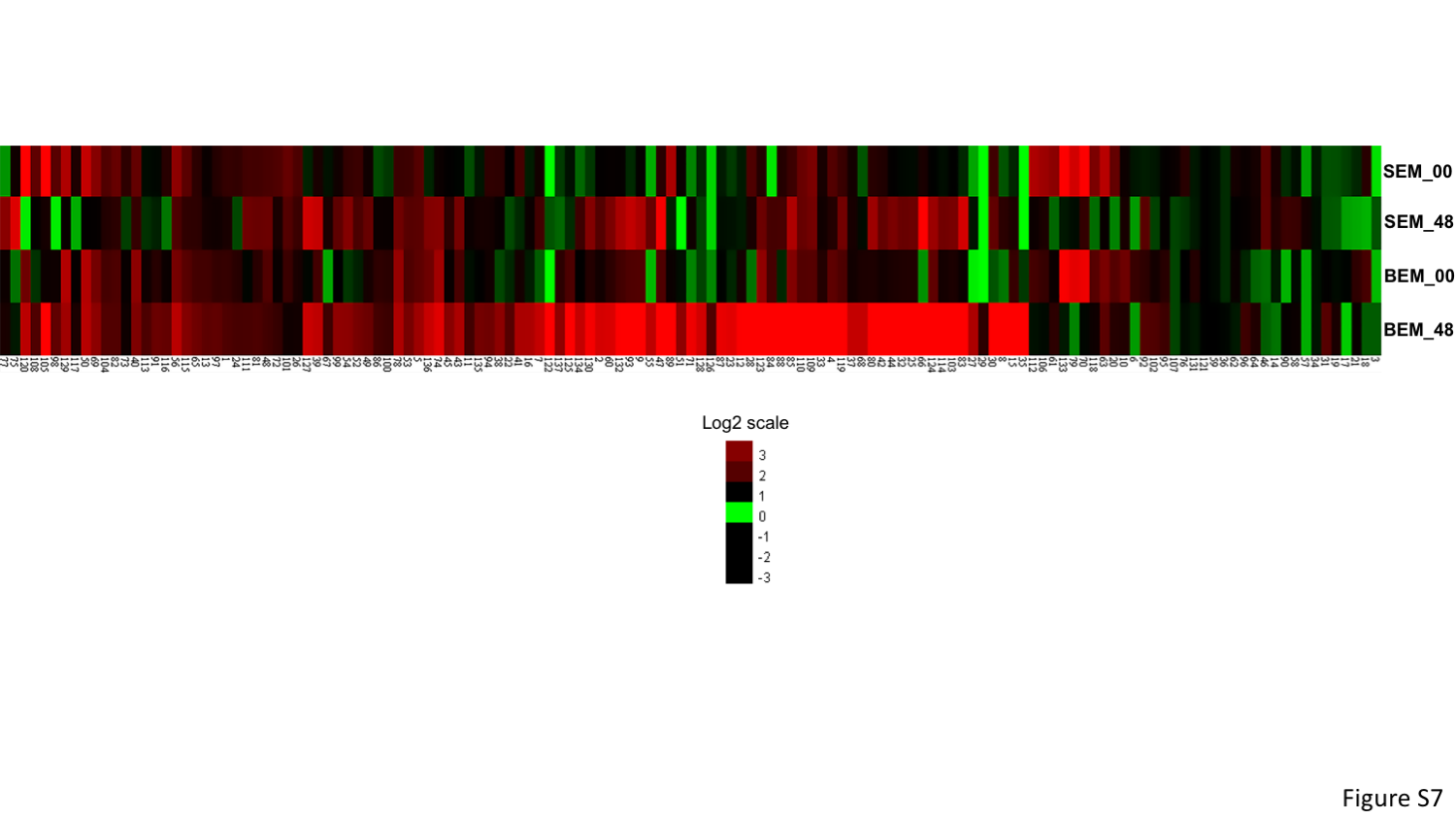


**Figure S7**: A comprehensive heat map of all the significant amino acids found in this study. The black color shows low abundance, red color shows high abundance, and green color shows equal abundance of metabolites between different samples. The number of different amino acids in the heat map corresponds to Supplementary Table S5.
